# Scenarios for handling the impact of COVID-19 based on food supply network through regional food hubs under uncertainty

**DOI:** 10.1016/j.heliyon.2020.e05128

**Published:** 2020-09-30

**Authors:** Tomy Perdana, Diah Chaerani, Audi Luqmanul Hakim Achmad, Fernianda Rahayu Hermiatin

**Affiliations:** a Department of Agro Socio-Economics, Faculty of Agriculture, Universitas Padjadjaran Jl.Raya Bandung-Sumedang KM 21, Jatinangor, Sumedang, West Java Province 45363, Indonesia; b Department of Mathematics, Faculty of Mathematics and Natural Sciences, Universitas Padjadjaran, Indonesia; c Agricultural Logistics and Supply Chain System (AGRILOGICS), Universitas Padjadjaran, Indonesia

**Keywords:** Food security, Regional food hubs, COVID-19 pandemic, Multi-objective, Many-to-many location-routing problem, Robust optimization, Systems theory, Systems engineering, Agricultural policy, Sustainable business, Logistics

## Abstract

This paper discusses an optimization model for handling the impact of the COVID-19 pandemic based on food supply network through regional food hubs (RFHs) under uncertainty. To this end, uncertainty is assumed in the demand and production data. During the Pandemic COVID-19 period, uncertainty has increased and the food supply chain system has changed. Thus, a new configuration of the food supply network requires analysis. In this paper, the concept of RFH is introduced to connect producers in rural areas and customers in urban areas. This paper determines the location and capacity of RFHs, the food supply network, the sum of maximum food supplies, and minimum logistics cost. This is done via a Multi-Objective Many-to-Many Location-Routing Problem model. Furthermore, since the conditions of the COVID-19 pandemic is uncertain, robust optimization is employed to handle uncertainties. During the current pandemic, red zones are defined to indicate the severity of the pandemic in a region. In this paper, the numerical experiment is considered for three scenarios: when a region is in large-scale social distancing, partial social distancing, or normal conditions. This social distancing situation is based on the defined red zones. The optimal food supply network is obtained for the three scenarios and the best scenario among the three is identified.

## 1 Introduction

The Indonesian government's initial attempt to minimize the COVID-19 pandemic's seriousness by taking early preventative measures has resulted in it forfeiting the opportunity to keep the COVID-19 pandemic under control in the short phase of the outbreak. According to Statistics Indonesia (2020), approximately 56.70% of Indonesia's population is in urban areas, and most COVID-19 cases in the country have been concentrated in these areas. Furthermore, the pandemic is having several effects on environmental, social, and economic aspects, as well as on food security.

One result of the COVID-19 pandemic is the bullwhip effect of food supply, which has caused uncertainty in demand and supply and increased the complexity of the food supply chain (Mitchell et al., 2020; Chen et al., 2020). As a result, the consumption of food in urban areas will be more volatile, which the distribution system will need to accommodate. During the pandemic, the emerging stockpile of staple food must gain significant attention as a government concern, specifically for areas designated as red zones^1^.

Thus, in the red zone, large-scale social distancing and restrictions are imposed, which impacts the distribution of some food products from the production areas. Large-scale social distancing also affects economic growth, changes consumer behavior, and results in the decrease in purchasing power parity for food products (Hobbs, 2020; Mukhamedjanova, 2020; Gregorioa and Ancog, 2020). This situation is also exacerbated by the closure of several public facilities, such as restaurants, cafés, schools, offices, and shopping centers. Public discourse in Indonesia is predictable focused on how the community can survive in the middle of large-scale social distancing and how people can get food supply (Rizou et al., 2020; Hobbs, 2020; Pulighe and Lupia, 2020).

These conditions have implications for the availability of food, hampered access to food for the community, restrictions on interaction between red zones and other areas, and the affordability of supplies (Pulighe and Lupia, 2020). The food distribution system is the foundation to decrease hunger, malnutrition, and food insecurity (Galanakis, 2020; Devereux et al., 2020). In recent times, food access has impacts on social, economic, and sustainability factors (Black et al., 2015; Hilmers et al., 2012; Walker et al., 2010). Therefore, understanding the complex system of food security is essential for social development (Béné, 2020; Devereux et al., 2020).

Several actions and policies have been implemented to address food security (Ge et al., 2018; Blay-Palmer et al., 2013; Johnson et al., 2012). Affordability, availability, and food accessibility for the communities has been the focus for the government, the private sector, and other stakeholders. Food security during the pandemic should be ensured by using technology such as e-commerce and the Internet of Things (IoT), not only to improve food security but to also help enhance food safety (Deng et al., 2015). Recovering downstream processing could help with adopting the latest technology (Galanakis, 2013), which can assist in providing food for the community and improving the value of the products (Galanakis, 2012; Hobbs, 2020; Barba et al., 2015). However, innovation, technology, and regulations are not keeping pace with the food consumption needs. Various problem in this regard have been apparent in recent time, such as uncertainty of food demand and supply, the high cost and inefficiency of logistics and delivery services, the high volume of losses and waste, the powerless of the upstream actors, and food safety (Mitchell et al., 2020; Chen et al., 2020; Rizou et al., 2020).

The COVID-19 pandemic has led to the emergence of a new era of the food supply network (Rizou et al., 2020) where food safety has become one of the focus components (Galanakis, 2020). To minimize the transmission of the coronavirus, the main concern must be product and worker hygiene to achieve food safety (WHO, 2020; Galanakis, 2020; Rizou et al., 2020). During the distribution process, the transportation system becomes an essential element to protect customers and food supply services providers (Gray, 2020). Attention to macromolecules and micromolecules could be given for the application of food safety and food hygiene protocols (Galanakis, 2015).

The pandemic has also changed consumer behavior in choosing food. Consumers seek to protect themselves and improve their immune system, and are concerned about changing their food consumption habits, bioactive ingredients, and nutritional content (Galanakis, 2020; Rizou et al., 2020; Zinoviadou et al., 2015).

Therefore, the food system should develop structured managerial systems, adequate infrastructure, facilities, and sustainable food supply (Galanakis, 2012, 2020; Rizou et al., 2020; Gray, 2020; Mitchell et al., 2020). An effective strategy to ensure food supply is to develop food hubs. A food hub is a business organization that supports local and regional producers, principally as an aggregator, distributor, manager of food security, and marketing operator for smallholder distributors, as well as to satisfy the wholesaler, supermarket, HORECA (Hotel, Restaurant, and Catering), and other marketplaces (Levkoe et al., 2018; Fischer et al., 2015). The conceptual model of food hubs is evolving, and hubs have widely adopted innovation to support locales and their goals (Ballantyne-Brodie and Telalbasic, 2017). In recent decades, regional food supply chains have become an essential issue, and food hubs are emerging as principal factors for developing viable local and regional food systems (Sharpe et al., 2020; Blay-Palmer et al., 2013).

Besides being a principal factor of the food system, Regional Food Hubs (RFHs) have a role in regulating and guaranteeing food supply, in ensuring food security and access in some regions, and will likely have a positive impact in the development of the regional economy (Sharpe et al., 2020; Etemadnia et al., 2015; Farmer and Betz, 2016; Kotani et al., 2020). For food security issues, the RFH becomes a critical facility to optimize food supply to consumers, as well as pay attention to sustainability aspect as a part of food security (Sharpe et al., 2020; Sonnino et al., 2014; Etemadnia et al., 2015; Farmer and Betz, 2016). RFH is a centralized facility with a business process management that aims to ensure food supplies and connect the various actors to strengthen food security and food safety through coverage of specific areas. RFH plays an essential role in developing local food systems and promotes entrepreneurship, develops local jobs, and fights food insecurity (O'Hara, 2015; Silver et al., 2017; Rose, 2017).

The RFH has become a network to collaborate with local producers and customers, forming local food networks by adopting socio-technical processes to address socio-economic and political tensions and support a fairer, more sustainable, and socially just food system (Prost et al., 2018). RFHs should develop through a mutually beneficial collaboration network by involving the Local Food Hub (LFH) to provide a source of the food supply. The LFH acts as a local aggregator hub for some local sources and specialty food distributors. It serves high-quality and locally grown products from small and mid-sized farms, and aims to meet the demands of its customer base (Barham et al., 2012).

Meanwhile, supermarkets, restaurants, cafés, and catering services partner with the RFH to sell products to the customers and other food service providers. RFH uses design-driven innovations to approach daily challenges, such as maintaining the constant balance of food availability in a region. RFH used as a continuous cycle of a strategy to change the sustainable local food supply networks system (Ballantyne-Brodie and Telalbasic, 2017). The food supply network system is a strategically interconnected network that improves the optimization, efficiency, and effectiveness of the food production and delivery process (Robinson et al., 2016). In the system, transparent edges between producers and consumers are a critical aspect to meet the sustainability goals (Brinkley, 2018; Le Velly and Dufeu, 2016).

The food supply network system must be prepared for various conditions, and must function not only under regular conditions but also during the pandemic. The RFH makes an essential contribution to the disaster relief supply by providing essential food for the communities during disasters and post disasters. In the time of pandemics, such as today, RFH could also function as a Food Resilience Network (FRN) to make food available, affordable, accessible, and hygienic for communities affected by the pandemic (Brand et al., 2019; Berno, 2017; Dasaklis et al., 2012). The pandemic might raise food deficiency due to the obstruction of the food supply chain.

The locations for the development of RFHs and optimal logistics for the food network must be determined based on various general conditions and, at the time of a pandemic, by considering the entire community as victims affected by it. The COVID-19 pandemic will have a long-lasting effect on the food supply network, including the development of e-commerce and customer preference toward local food (Hobbs, 2020; Galanakis, 2020).

Research on RFH logistics optimization is largely underway in Europe, the United States, Canada, and Mexico (Mittal et al., 2018). The conceptual model of RFH is still emerging and is increasingly applied to the development of logistics systems for agriculture (Mittal and Krejci, 2019; Fischer et al., 2015). The RFH movement embraces a bank of locally adapted food networks, food traceability, inventory, and warehouse operation management (Mittal and Krejci, 2019; Prost et al., 2018). Further, RFH has been developed for the sustainability of the local food system (Cleveland et al., 2014). Sharpe et al. (2020) notes the importance of providing access to healthy food for all levels of society by optimizing the role of food hubs. Other research has developed conceptual models for optimizing food supply networks during the pandemic (Liu et al., 2020; Tang and Lau, 2013; Dasaklis et al., 2012). However, studies have not considered the location for the development of RFH and the food network in all conditions, especially during the pandemic.

This paper aims to determine the optimal location of RFH and food supply networks connecting producers in rural areas and customers in urban areas. It focuses on food security to ensure that food is available, affordable, accessible, and safe at all times, including in the new era food supply network.

The Many-to-Many Location-Routing Problem (MMLRP) is used in this paper to examine the location and capacity of RFH, the food supply network, the sum of maximum food supplies, and minimum logistics costs. Several pervious works have attempted to solve the food supply network problem using MMLRP. Wang et al. (2018) discuss MMLRP for cold chain logistics. The scheme proposed in their model consists of supply point, Distribution Center (DC), and demand point. In their model, MMLRP is used to determine DC location and the network distribution. Another work of MMLRP in the food supply network problem is Hiassat et al. (2017), who use the MMLRP model for perishable products and propose a scheme that consists of warehouse and customer. MMLRP is used in their model to determine the warehouse location and food distribution network. However, neither model considers uncertainties, such as demand or production capacity uncertainties. They also do not consider multi-item products in their MMLRP model.

Therefore, an MMLRP model that considers multi-item (multi-commodity) products is developed in this paper. This model also addressed several uncertainties, such as food demand, food production, and distribution cost uncertainties using robust optimization (RO) that assumes that the uncertain parameters lie in an uncertainty set (Ben-Tal et al., 2009; Gorissen et al., 2015; Yanıkoğlu et al., 2019). RO aims to remove all the uncertain parameters from the uncertain problem and obtain a robust reformulation of the uncertain problem called a Robust Counterpart (RC). Furthermore, a two-objective MMLRP is proposed in this paper: (1) maximize food supply, and (2) minimize logistics cost. The latter includes food handling costs under several health protocols to guarantee food safety. To solve this Multi-Objective MMLRP (MOMMLRP), the lexicographic method is applied. The lexicographic method is a way to handle multi-objective optimization problems, by priority-ordering the objectives and solving them iteratively (Stanimirovic, 2012).

The rest of this paper is organized as follows: section 2 paper discusses the problem statement related to this paper, followed by the methodology employed in this research. It is followed by a case study that explains the research location. The results and discussion sections describe three alternative simulation scenarios in the COVID-19 situation. The conclusion focuses on the result of the paper that answer the problems discussed in the next section.

## 2 Problem statement

This paper addresses the problem of determining the optimal location of RFH by considering aspects of sustainability and food security for all conditions, including during pandemics. By connecting the local producer and customers through RFH, the food supply network could be developed to maintain food security. This paper discusses three alternative scenarios to tackle RFH during the pandemic. It aims to identify the best alternative RFH location and capacity and food distribution network; the maximum product fulfillment of each region; and the minimum logistic costs in red zones, defined as an area at the epicenter COVID-19 infection.

**Figure 1:**
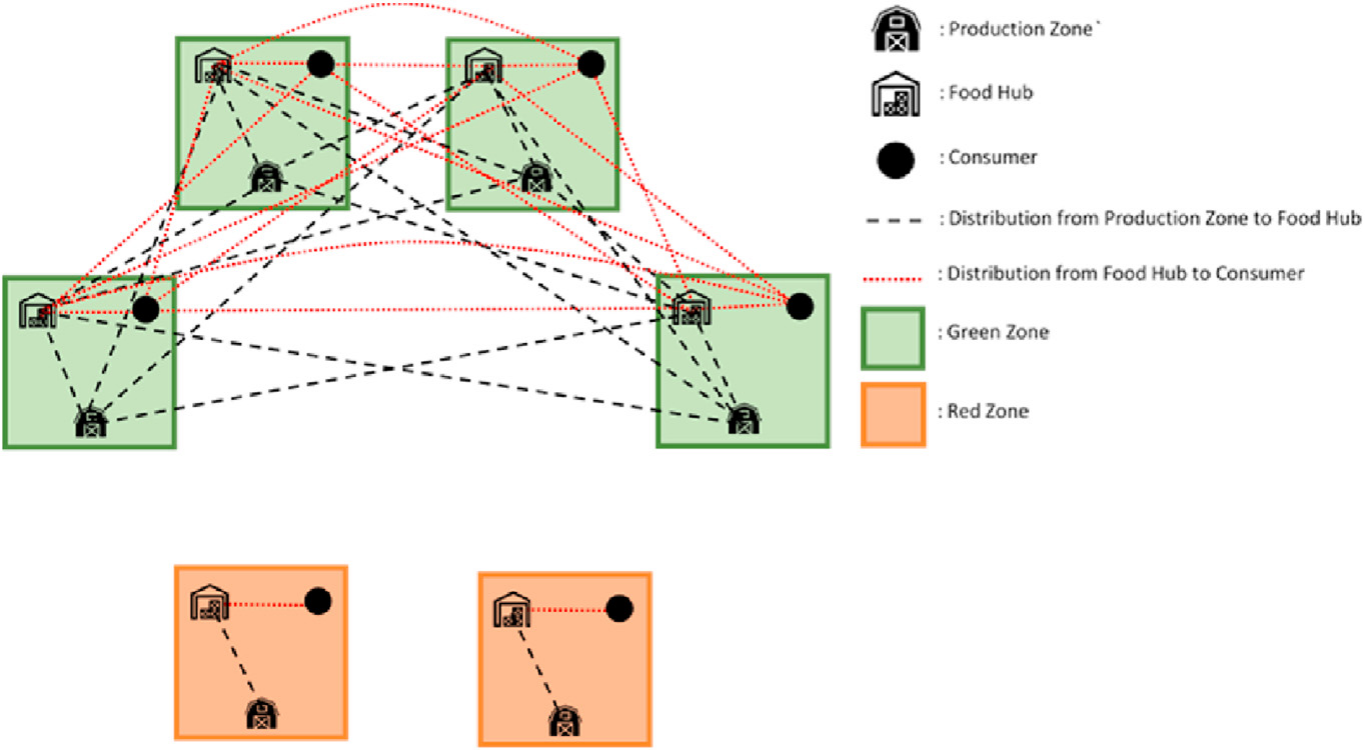
Food distribution problem of scenario 1.

Scenario 1 represents the large-scale social distancing condition in red zone areas. Food supply from the green zone is not allowed in the red zone. The red zone must survive by relying on the food products of its region. This condition is intended to minimize the spread of the COVID-19 pandemic and to determine if the red zone can fulfill food demand. Further, this condition is designed to determine if the green areas can meet food demand without any supplies from the red zones. The food distribution problem in scenario 1 is illustrated in Figure 1.

**Figure 2:**
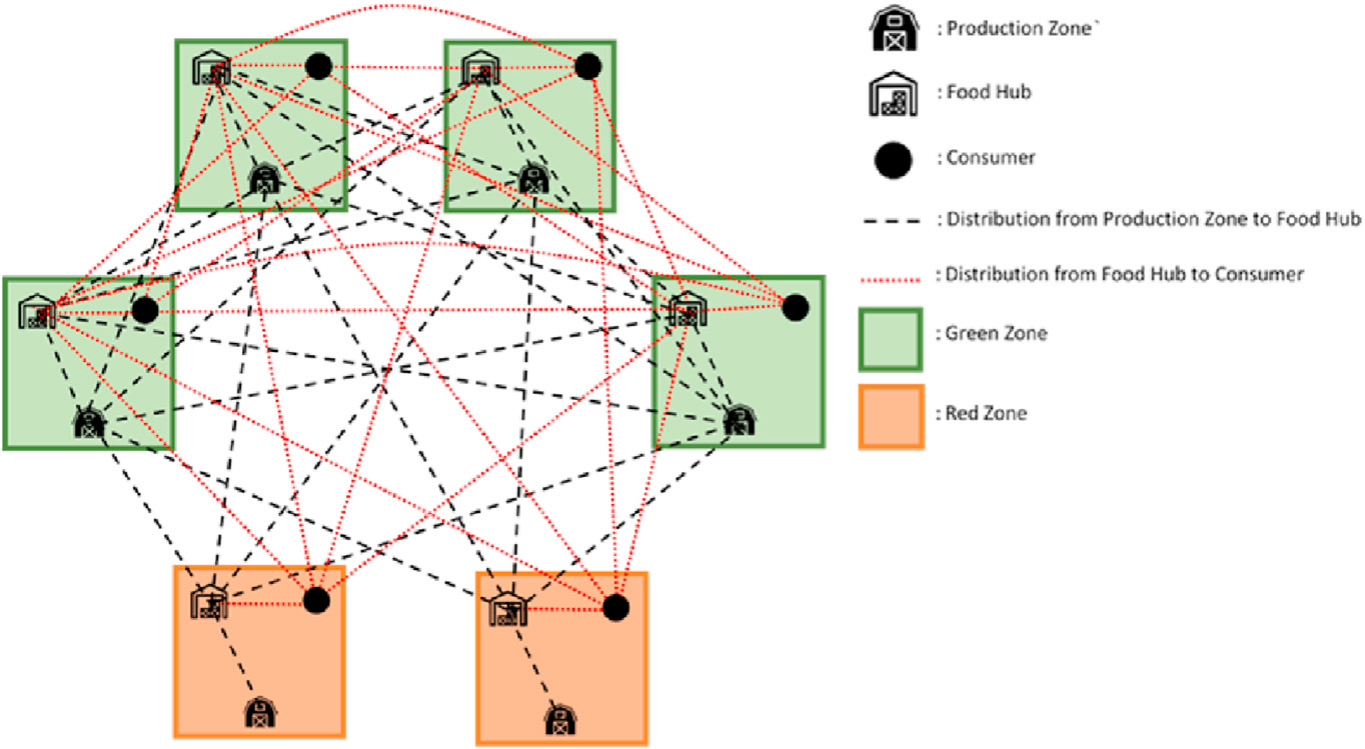
Food distribution problem of scenario 2.

In scenario 2, the partial social distancing condition was applied in the red zone. These areas are forbidden from delivering food products to other regions. However, other regions can supply food to red zones. These conditions are meant to decrease the spreading of COVID-19 from the red zone to other areas, and other regions can support the red zones. This scenario problem is illustrated in Figure 2.

**Figure 3:**
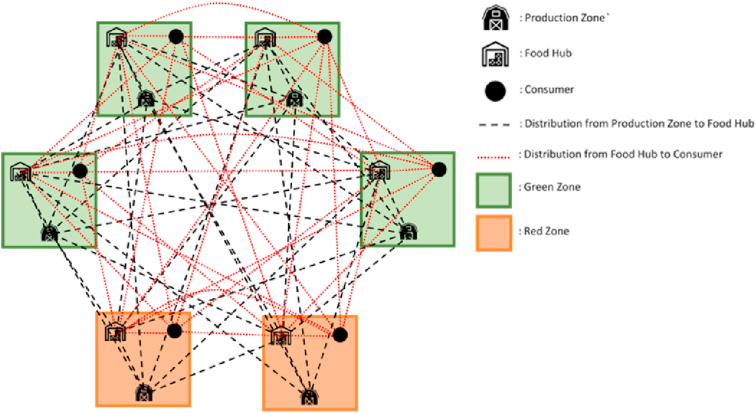
Food distribution problem of scenario 3.

Alternative scenario 3 is used in the new era condition in which a food supply network is allowed to function as usual. However, security protocols are applied following health standards for the prevention of COVID-19 due to the transformations in the food supply chain during this COVID-19 pandemic (Rizou et al., 2020). This scenario considers the function of RFH after the COVID-19 pandemic condition. This condition is intended to compare the results of the two preceding alternative scenarios. Figure 3 illustrates the problem in scenario 3.

An optimization model with MOMMLRP can describe the best alternative solution for complex problems. Variables, objective functions, constraints, and coefficients are used as the central concept of optimization models. The assumptions for the three alternative scenarios are determined using several parameters: food demand, production capacity, and operational costs. In this paper, the food in question is the commodity of rice as the staple food of Indonesians, chicken eggs as an alternative source of animal protein consumed, and vegetables as a source of fiber. In this study, vegetable commodities are limited to the most common vegetables consumed by Indonesians, shallots, red chilies, beans, potatoes, carrots, tomatoes, spinach, and water spinach.

Furthermore, each scenario's MOMMLRP model involves several uncertain parameters, that is, food demand, food production, and distribution cost, which makes the MOMMLRP model uncertain. RO is applied to solve the uncertain MOMMLRP by removing all uncertain parameters from the uncertain problem and to obtain a robust reformulation of the uncertain problem called the RC, which is used to give a robust alternative scenario. The RC of each scenario is solved by numerical simulation to find a robust solution that is feasible under all possible disruptions.

## 3 Methodology

General optimization problems often involve uncertain parameters. The uncertain parameters are likely caused by measurement error, such as temperature or size measurements. The uncertain parameters could also be caused by mistakes in estimating the data, for instance in estimating demand, profit, selling price, or travel time. In this paper, RO is used to handle the uncertain parameters, that is, food demand, production capacity, and distribution cost.

RO is a method to handle uncertain parameters that are assumed to be part of an uncertainty set (Ben-Tal and Nemirovski, 2002). The method's primary purpose is to achieve an optimal solution that is robust to uncertain data. The mathematical formulation for the general uncertain linear programming as proposed by Ben-Tal et al. (2009) and discussed by Gorissen et al. (2015) is given by (1).

(1) $$\underset{x}{\text{min}}\left\{c^{T}x:Ax\leq b,\forall \left(c,A,b\right)\in U,x\geq 0\right\},$$

where $c,x\in R^{n},A\in M_{m\times n}\left(R\right)$, $b\in R^{m}$, and U is primitive uncertainty set (an uncertainty set that has not yet been determined).

The aim of RO is to remove all the uncertain parameters in uncertain problem (1) and obtain a robust reformulation of it, called the RC. To solve the uncertain problem (1), several assumptions are necessary, as discussed by Ben-Tal et al. (2009), Gorissen et al. (2015), and Yanıkoğlu et al. (2019): (A1) the objective function is certain, (A2) the right-hand side vector is certain, and (A3) the uncertainty is constraint wise.

Under assumptions (A1) and (A2)—that is, assume that $c\in R^{n}$ and $b\in R^{m}$ are certain—the uncertain problem (1) can be rewritten as (2).

(2) $$\underset{x}{\text{min}}\left\{c^{T}x:Ax\leq b,\forall A\in U,x\geq 0\right\}.$$

Now, since $c\in R^{n}$ and $b\in R^{m}$ are certain, the robust reformulation of (1), which is generally referred to as the RC problem, is given by (3):

(3) $$\underset{x}{\text{min}}\left\{c^{T}x:A\left(\zeta \right)x\leq b,\forall \zeta \in Z,x\geq 0\right\}.$$

where $Z\subset R^{L}$.

The constraint in (3) can be rewritten constraint wise. Denoting $A_{i}$ as the i-th column in matrix A, (3) becomes (4):

(4) $${\left(A_{i}+D_{i}\zeta \right)}^{T}x\leq b_{i},\forall i\in \left\{1,2,\cdots ,m\right\},\forall \zeta \in Z,$$

where $A_{i}\in R^{n},D_{i}\in M_{n\times L}\left(R\right)$, and $b_{i}\in R,\forall i\in \left\{1,2,\cdots ,m\right\}$.

In this paper, a box uncertainty set is used, with uncertainty parameters as discussed by Ben-Tal et al. (2009) and Gorissen et al. (2015). The box uncertainty set is defined by $Z_{\text{box}}=\left\{\zeta :||\zeta |{|}_{\infty }\leq 1\right\}$ (Gorissen et al., 2015). Thus, (4) can be written as (5):

(5) $${\left(A_{i}+D_{i}\zeta \right)}^{T}x\leq b_{i},\forall i\in \left\{1,2,\cdots ,m\right\},\forall \zeta \in Z_{\text{box}}.$$

By using the concept of the best worst-case reformulation as discussed by Gorissen et al. (2015) thus:

$${\left(A_{i}+D_{i}\zeta \right)}^{T}x\leq b_{i},\forall i\in \left\{1,2,\cdots ,m\right\},\forall \zeta \in Z_{\text{box}}$$

$$\equiv {A_{i}}^{T}x+{\left(D_{i}\zeta \right)}^{T}x\leq b_{i},\forall i\in \left\{1,2,\cdots ,m\right\},\forall \zeta \in Z_{\text{box}}$$

(6) $$\equiv {A_{i}}^{T}x+\underset{\zeta :||\zeta |{|}_{\infty }\leq 1}{\text{max}}({D_{i}}^{T}x)\zeta \leq b_{i},\forall i\in \{1,2,\cdots ,m\}.$$

Take ζ as the unit vector:

(7) $$\zeta =\frac{{D_{i}}^{T}x}{\left|\right|{D_{i}}^{T}x\left|\right|},\forall i\in \left\{1,2,\cdots ,m\right\},$$

Thus, (6) can be rewritten as follows:

(8) $${A_{i}}^{T}x+||{D_{i}}^{T}x|{|}_{\infty }\leq b_{i},\forall i\in \{1,2,\cdots ,m\}\text{.}$$

In the case of box uncertainty, the RC of (5) is given by (9):

(9) $${A_{i}}^{T}x+||{D_{i}}^{T}x|{|}_{1}\leq b_{i},\forall i\in \{1,2,\cdots ,m\}\text{.}$$

By repeating this step for all other uncertain constraints, RC for the uncertain problem (3), where $\zeta \in Z_{\text{box}}$, is given by (10):

(10) $$\underset{x}{\text{min}}\left\{c^{T}x:{A_{i}}^{T}x+||{D_{i}}^{T}x|{|}_{1}\leq b_{i},x\geq 0|\forall i\in \left\{1,2,...,m\right\}\right\}.$$

## 4 Results and discussion

This section describes the experimental MOMMLRP model for optimal RFH locations and food networks based on the three scenarios described in the Problem Statement section. It also discusses the Robust MOMMLRP model to handle food demand, production capacity, and distribution cost uncertainties. The MOMMLRP models are designed based on the three scenarios to describe the development of an RFH location and network in West Java Province.

### 4.1 MOMMLRP model based on COVID-19 pandemic red zones scenarios

This subsection discussed the MOMMLRP model developed in this study for the three scenarios. First, the MOMMLRP model for large-scale social distancing is discussed. Then, the MOMMLRP model for partial social distancing and for normal conditions are discussed based on the differences from the large-scale social distancing model. The MOMMLRP model of the three scenarios is then summarized.

#### 4.1.1 MOMMLRP model for large-scale social distancing (Scenario 1)

To solve the problems in scenario 1, a MOMMLRP model seeks the maximum demand fulfillment and minimum total cost of logistics. The sets that are used in this model are I, which represents the demand zone, J, which represents RFH, K, which represents the production zone, C, which represents commodity, and R, which represents the red zone or the epicenter of COVID-19 infection.

The parameters used in scenario 1 are $d_{ci}$, which represents the demand for food c from district i (kilotonne/day); $v_{ci}$, which represents the selling price of commodity c in district i (Rp/kilotonne); $f_{ck}$, which represents the production capacity of commodity c on central production district k (kilotonne/day); $b_{ji}$, which represents distribution cost between district j and district i for transporting the food (Rp/kilotonne); q which represents food handling cost under several health protocols (Rp/kilotonne); and h which represents the RFH-building cost (Rp/hub).

The decision variables used in this model are $x_{j}$, which decides if the RFH will be built on district j, which takes 1 if the RFH will not be built on district j, and 0 otherwise; $P_{cj}$, which decides the RFH capacity for commodity c in district j (kilotonne/day); $y_{ckj}$, which decides how much of the commodity c produced on district k is sent to the RFH in district j in proportion to production capacity $f_{ck}$; and $w_{cji}$ which decides how much commodity demand c on district i is fulfilled by the RFH in district j, in proportion to demand $d_{ci}$. The decision variables $x_{j}$ are binary ($x_{j}\in 0,1,\forall j\in J$). The decision variables $P_{cj}$ are real numbers ($P_{cj}\in R,\forall c\in C,j\in J$). The decision variables $y_{ckj}$ are real numbers between 0 and 1 ($y_{ckj}\in \left[0,1\right],\forall c\in C,k\in K,j\in J$). Decision variables $w_{cji}$ are real numbers between 0 and 1 ($w_{cji}\in \left[0,1\right],\forall c\in C,j\in J,i\in I$).

This model has two objectives, maximizing demand fulfillment and minimizing logistics cost. The source of the logistics cost is the operational costs for distributing the food between segments (production area–RFH–customer). The logistics cost also includes the operational costs of the RFH building and food handling under the pandemic with several precautions to maintain food safety.

The objective function to maximize the demand fulfillment is given by (11):

(11) $$\max\left\{\underset{c\in C}{\sum }\underset{i\in I}{\sum }v_{ci}\underset{j\in J}{\sum }w_{cji}\right\}$$

The objective function to minimize the logistics costs as explained above is given by (12):

(12) $$\min\left\{h\underset{j}{\sum }x_{j}+\underset{c\in C}{\sum }\underset{j\in J}{\sum }\underset{i\in I}{\sum }b_{ji}d_{ci}w_{cji}+q\underset{c\in C}{\sum }\underset{j\in J}{\sum }P_{cj}+\underset{c\in C}{\sum }\underset{k\in K}{\sum }\underset{j\in J}{\sum }b_{kj}f_{ck}y_{ckj}\right\}$$

The constraints that guarantee the determined RFH capacity of all commodities are determined based on the demand and the production capacity are given by (13):

(13) $$\underset{k\in K}{\sum }f_{ck}y_{ckj}=P_{cj},\forall c\in C,j\in J$$

$$\underset{i\in I}{\sum }d_{ci}w_{cji}=P_{cj},\forall c\in C,j\in J$$

The constraint that guarantees that the commodities delivered from the production area to the RFH will not exceed the production capacity for those commodities is given by (14):

(14) $$\underset{j\in J}{\sum }y_{ckj}\leq 1,\forall c\in C,k\in K$$

The constraint that guarantees that the demand fulfilled by the RFH will not exceed the demand is given by (15):

(15) $$\underset{j\in J}{\sum }w_{cji}\leq 1,\forall c\in C,i\in I$$

The following constraint guarantees that no commodities will be delivered to the red zone if the RFH is not built in that zone:

(16) $$y_{ckj}\leq x_{j},\forall c\in C,k\in K,j\in J$$

The constraint that guarantees that no zone's demand will be fulfilled if the RFH will not be built in that zone is given by (17):

(17) $$w_{cji}\leq x_{j},\forall c\in C,j\in J,i\in I$$

The constraint given below guarantees that there will be no food distribution to an RFH in the red zone from the production area in other zones:

(18) $$y_{ckr}=0,\forall k\in K-\left\{r\right\},c\in C,r\in R$$

The following constraint guarantees that there will be no food distribution to the customer in the red zone from an RFH outside the red zone:

(19) $$w_{cjr}=0,\forall j\in J-\left\{r\right\},c\in C,r\in R$$

The constraint that guarantees that there will be no food distribution from a production area in the red zone to an RFH in other zones is given by (20):

(20) $$y_{crj}=0,\forall j\in J-\left\{r\right\},c\in C,r\in R$$

The following constraint guarantees that there will be no food distribution from an RFH in the red zone to customers in other zones:

(21) $$w_{cri}=0,\forall i\in I-\left\{r\right\},c\in C,r\in R$$

#### 4.1.2 MOMMLRP model for partial social distancing (Scenario 2)

The difference between the first and second scenarios is that in the second, the production areas would distribute food to the RFHs in red zones. This is considered so that the food demand in the red zones can be fulfilled, aiming to decrease food insecurity. However, the quantity of food distributed to the red zone is limited by the maximum transportation capacity for food per delivery period. Accordingly, parameter $n_{c}$ has been added, which represents the maximum amount of commodity c that could be distributed in one-way distribution (Tonne/distribution). Also, two new decision variables are introduced, $m_{2r}$ and $m_{4r}$. Decision variable $m_{2r}$ decides how many times the distribution should be done to the RFH in red zone r. Decision variable $m_{4r}$ decides how many times the distribution should be done to the consumer in red zone r.

Constraint (18) in Scenario 1 is changed to constraint (22) below, which guarantees that the food distribution to the RFH in red zone is allowed. Constraint (22) also calculates how many times the distribution should be done, as follows:

(22) $$\underset{k\in K-\left\{r\right\}}{\sum }y_{ckr}f_{ck}\leq m_{2r}n_{c},\forall c\in C,r\in R$$

Constraint (19) in Scenario 1 is modified into constraint (23) below, which ensures that the food distribution to consumers in the red zone is allowed. Constraint (23) also calculate how many times the distribution should be done, as follows:

(23) $$\underset{j\in J-\left\{r\right\}}{\sum }w_{cjr}d_{cr}\leq m_{4r}n_{c},\forall c\in C,r\in R$$

#### 4.1.3 MOMMLRP model for the new era food supply network (Scenario 3)

Scenario 3 adopts a typical food supply network, in which there are no constraints on the distribution process of each zone aiming to fulfill the food demand. The limit on scenario 3 is the maximum capacity of food supply delivery in one distribution period, noted by $n_{c}$ (Tonne/distribution). Corresponding to the statement above, two new decision variables are added, $m_{1r}$ and $m_{3r}$. Decision variable $m_{1r}$ decides how many times the distribution should be done from the red zone r. Decision variable $m_{3r}$ decides how many times the distribution should be done from the RFH in red zone r.

Constraint (20) in Scenario 1 was modified into constraint (24). Constraint (24) guarantees that the food distribution from the red zone is allowed. Constraint (24) also calculates how many times the distribution should be done as follows:

(24) $$\underset{j\in J-\left\{r\right\}}{\sum }y_{crj}f_{cr}\leq m_{1r}n_{c},\forall c\in C,r\in R$$

Constraint (21) in Scenario 1 was modified into constraint (25), which guarantees that the food distribution from RFHs in red zones is allowed. Constraint (25) also calculate how many times the distribution should be done as follows:

(25) $$\underset{i\in I-\left\{r\right\}}{\sum }w_{cri}d_{ci}\leq m_{3r}n_{c},\forall c\in C,r\in R$$

The differences between the models in the three scenarios are measured based on the maximum, minimum, and constraints used in each scenario. The differences between the MOMMLRP model of the three scenarios are summarized in Table 1.

**Table 1:** The MOMMLRP model for each scenario.

	Scenario 1	Scenario 2	Scenario 3
Maximize	(11)	(11)	(11)
Minimize	(12)	(12)	(12)
Constraints	(13), (14), (15), (16), (17), (18), (19), (20), (21)	(13), (14), (15), (16), (17), (20), (21), (22), (23)	(13), (14), (15), (16), (17), (22), (23), (24), (25)

### 4.2 Robust model formulation

In this study, we assume that the uncertain parameters are demand for the commodities $d_{ci}$, production capacity for commodity $f_{ck}$, and transportation costs between locations $b_{kj}$ and $b_{ji}$.The multiplication of the uncertain parameters $b_{ji}d_{ci}$ in objective function (12) is considered as a single uncertain parameter $m_{cji}$, which represents the maximum travelling cost to fulfill the demand for commodity c in district i from the RFH in district j. Besides, the multiplication of the uncertain parameters $b_{kj}f_{ck}$ in objective function (12) is considered as a single uncertain parameter $n_{ckj}$, which represents the maximum travelling cost to deliver all the commodity c that was produced in district k to the RFH in district j. The transformation is given as follows:

(26) $$b_{ji}d_{ci}=m_{cji},\forall c\in C,j\in J,i\in I$$

$$b_{kj}f_{ck}=n_{ckj},\forall c\in C,k\in J,j\in J$$

Thus, the uncertain parameters considered in this model are $d_{ci},f_{ck},m_{cji},n_{ckj}$, as in (27):

(27) $$\forall \left\{f_{ck},d_{ci},m_{cji},n_{ckj}\right\}\in U$$

By substituting (26) into objective function (12), the objective function (12) a new objective function (28) is obtained:

(28) $$\min\left\{h\underset{j\in J}{\sum }x_{j}+\underset{c\in C}{\sum }\underset{j\in J}{\sum }\underset{i\in I}{\sum }m_{cji}w_{cji}+q_{c}\underset{c\in C}{\sum }\underset{j\in J}{\sum }P_{cj}+\underset{c\in C}{\sum }\underset{k\in K}{\sum }\underset{j\in J}{\sum }n_{ckj}y_{ckj}\right\}$$

The objective function (12) is replaced with (28). The uncertain MOMMLRP model for each scenario is presented in Table 2.

**Table 2:** The uncertain MOMMLRP model for each scenario.

	Scenario 1	Scenario 2	Scenario 3
Maximize	(11)	(11)	(11)
Minimize	(28)	(28)	(28)
Constraints	(13), (14), (15), (16), (17), (18), (19), (20), (21), (27)	(13), (14), (15), (16), (17), (20), (21), (22), (23), (27)	(13), (14), (15), (16), (17), (22), (23), (24), (25), (27)

RO is employed to handle the uncertain problem. This can be done by removing all uncertain parameters from the uncertain problem to obtain a robust reformulation of the uncertain problem called the RC, as discussed in the methodology section. By using the assumption (A1), the uncertain objective function (28) becomes (29):

(29) $$\min\left\{z+h\underset{j\in J}{\sum }x_{j}+q_{c}\underset{c\in C}{\sum }\underset{j\in J}{\sum }P_{cj}\right\}$$

with a new decision variable z added and defined in a new constraint:

(30) $$\underset{c\in C}{\sum }\underset{j\in J}{\sum }\underset{i\in I}{\sum }m_{cji}w_{cji}+\underset{c\in C}{\sum }\underset{k\in K}{\sum }\underset{j\in J}{\sum }n_{ckj}y_{ckj}\leq z$$

All the uncertain parameters are on the left-hand side of constraints. The uncertain constraints in the uncertain MOMMLRP model for all scenarios are (13) and (30). By using assumption (A3), the uncertain MOMMLRP can be used for every single uncertain constraint (13) and (30). The single uncertain constraint (13) and (30) can be written in vector as given in (31) and (32):

(31) $$f^{T}y=P$$

$$d^{T}w=P$$

(32) $$m^{T}w+n^{T}y\leq z$$

where $f\in R^{\left|K\right|},d\in R^{\left|I\right|},m\in R^{\left|C\right|+\left|J\right|+\left|I\right|},n\in R^{\left|C\right|+\left|K\right|+\left|J\right|}$ and P,z∈R.

As it is assumed that the parameter f,d,m and n are contained in a box uncertainty set, the constraints (31) and (32) can be rewritten as (33) and (34):

(33) $${\left(f+D_{1}\zeta_{1}\right)}^{T}y=P$$

$${\left(d+D_{2}\zeta_{2}\right)}^{T}w=P$$

(34) $${\left(m+D_{3}\zeta_{3}\right)}^{T}w+{\left(n+D_{4}\zeta_{4}\right)}^{T}y\leq z$$

Then, the RC of (33) and (34) are given by (35) and (36):

(35) $$f^{T}y+{\mathrm{||D}}_{1}^{T}y{||}_{1}=P$$

$$d^{T}w+{\mathrm{||D}}_{2}^{T}y{||}_{1}=P$$

(36) $$m^{T}w+{\mathrm{||D}}_{3}^{T}w{||}_{1}+n^{T}y+{\mathrm{||D}}_{4}^{T}y{||}_{1}\leq z$$

In this case, $D_{1},D_{2},D_{3}$, and $D_{4}$ are diagonal matrices. Thus, the terms of ${D_{1}}^{T}y,{D_{2}}^{T}w,{D_{3}}^{T}w$, and ${D_{4}}^{T}y$ are vectors as presented in (37) and (38):

(37) $$D_{1}^{T}y=\left[\begin{array}{ccccc} s_{1} & 0 & 0 & \cdots & 0\\ 0 & s_{2} & 0 & \cdots & 0\\ \vdots & \vdots & \vdots & \vdots & \vdots \\ 0 & 0 & 0 & \cdots & s_{\left|K\right|} \end{array}\right]\left[\begin{array}{c} y_{1}\\ y_{2}\\ \vdots \\ y_{\left|K\right|} \end{array}\right],D_{2}^{T}\text{w}=\left[\begin{array}{ccccc} t_{1} & 0 & 0 & \cdots & 0\\ 0 & t_{2} & 0 & \cdots & 0\\ \vdots & \vdots & \vdots & \vdots & \vdots \\ 0 & 0 & 0 & \cdots & t_{\left|I\right|} \end{array}\right]\left[\begin{array}{c} w_{1}\\ w_{2}\\ \vdots \\ w_{\left|I\right|} \end{array}\right]$$

(38) $$D_{3}^{T}w=\left[\begin{array}{ccccc} u_{1} & 0 & 0 & \cdots & 0\\ 0 & u_{2} & 0 & \cdots & 0\\ \vdots & \vdots & \vdots & \vdots & \vdots \\ 0 & 0 & 0 & \cdots & u_{\left|C\right|+|J\}+|I|} \end{array}\right]\left[\begin{array}{c} w_{1}\\ w_{2}\\ \vdots \\ w_{\left|C\right|+\left|J\right|+\left|I\right|} \end{array}\right],\;D_{2}^{T}\text{y}=\left[\begin{array}{ccccc} v_{1} & 0 & 0 & \cdots & 0\\ 0 & v_{2} & 0 & \cdots & 0\\ \vdots & \vdots & \vdots & \vdots & \vdots \\ 0 & 0 & 0 & \cdots & v_{\left|C\right|+\left|K\right|+\left|J\right|} \end{array}\right]\left[\begin{array}{c} y_{1}\\ y_{2}\\ \vdots \\ y_{\left|C\right|+\left|K\right|+\left|J\right|} \end{array}\right]$$

Since the $\ell^{1}$-norm of those vectors are the summation of its elements, constraint (35) and (36) can be rewritten in the form of a summation as given in (39) and (40):

(39) $$\underset{k\in K}{\sum }\bar{f_{ck}}y_{ckj}+\underset{k\in K}{\sum }s_{ck}y_{ckj}=P_{cj},c\in C,j\in J$$

$$\underset{i\in I}{\sum }\bar{d_{ci}}w_{cji}+\underset{i\in I}{\sum }t_{ci}w_{cji}=P_{cj},c\in C,j\in J$$

(40) $$\underset{c\in C}{\sum }\underset{j\in J}{\sum }\underset{i\in I}{\sum }\bar{m_{cji}}w_{cji}+\underset{c\in C}{\sum }\underset{j\in J}{\sum }\underset{i\in I}{\sum }u_{cji}w_{cji}+\underset{c\in C}{\sum }\underset{k\in K}{\sum }\underset{j\in J}{\sum }\bar{n_{ckj}}y_{ckj}+\underset{c\in C}{\sum }\underset{k\in K}{\sum }\underset{j\in J}{\sum }v_{ckj}y_{ckj}\leq z$$

where $\bar{f_{ck}}$ is the average production capacity for commodity c in production area k; $\bar{d_{ci}}$ is the average demand for commodity c in district i; $\bar{m_{cji}}$ is the average distribution cost to fulfill the demand for commodity c in district i from the RFH in district j; $\bar{n_{ckj}}$ is the average distribution cost to deliver all the commodity c that was produced in district i to the RFH in district j; and $s_{ck},t_{ci},u_{cji},v_{ckj}$ is the uncertain part of $\bar{f_{ck}},\bar{d_{ci}},\bar{m_{cji}},\bar{n_{ckj}}$. In this paper, all the uncertain parts are set based on the average.

By repeating the same step for all other uncertain constraints, the RC of uncertain constraint (13) and (30) is given in (41) and (42):

(41) $$\underset{k\in K}{\sum }\bar{f_{ck}}y_{ckj}+\underset{k\in K}{\sum }s_{ck}y_{ckj}=P_{cj},\forall c\in C,j\in J$$

$$\underset{i\in I}{\sum }\bar{d_{ci}}w_{cji}+\underset{i\in I}{\sum }t_{ci}w_{cji}=P_{cj},\forall c\in C,j\in J$$

(42) $$\underset{c\in C}{\sum }\underset{j\in J}{\sum }\underset{i\in I}{\sum }\bar{m_{cji}}w_{cji}+\underset{c\in C}{\sum }\underset{j\in J}{\sum }\underset{i\in I}{\sum }u_{cji}w_{cji}+\underset{c\in C}{\sum }\underset{k\in K}{\sum }\underset{j\in J}{\sum }\bar{n_{ckj}}y_{ckj}+\underset{c\in C}{\sum }\underset{k\in K}{\sum }\underset{j\in J}{\sum }v_{ckj}y_{ckj}\leq z$$

The RC of the uncertain MOMMLRP for each scenario is presented in Table 3. The RC model of all scenarios in Table 3 is then called the Robust MOMMLRP model (RMOMMLRP), which is robust against the uncertain food demand, production capacity, and distribution cost.

**Table 3:** Robust Counterpart of MOMMLRP model for each scenario.

	Scenario 1	Scenario 2	Scenario 3
Maximize	(11)	(11)	(11)
Minimize	(29)	(29)	(29)
Constraints	(14), (15), (16), (17), (18), (19), (20), (21), (41), (42)	(14), (15), (16), (17), (20), (21), (22), (23), (41), (42)	(14), (15), (16), (17), (22), (23), (24), (25), (41), (42)

The RMOMMLRP models have two objectives as seen in Table 3, which maximizing demand fulfillment and minimizing logistics cost. Therefore, the lexicographic method is used to solve the problem. The lexicographic method is a way to handle multi-objective optimization problem (Stanimirovic, 2012). It orders the objectives based on priority (Rao, 2019). The multi-objective problem is then solved iteratively from the first objective and substituted back to the problem as a new constraint with the following objective.

### 4.3 Case study

Case studies are carried out in West Java, the most populous province in Indonesia. According to Statistics Indonesia (2020), West Java is the third leading agricultural producer in Indonesia after East Java and Central Java, as well as a strategic marketing area because 18.67% of Indonesia's population is in West Java Province. In this study, the commodities analyzed are the strategic commodities of the people of West Java, that is, rice, chicken eggs (particular chicken eggs), and vegetables. Rice as a staple food for Indonesia as a complex carbohydrate source. Chicken eggs (layer) are the most consumed protein due to the low price and availability.

Moreover, vegetables are considered a high-value commodity, that is, shallots, red chilies, beans, potatoes, carrots, tomatoes, spinach, and water spinach. The demand and production capacity of each commodity are assumed to be uncertain. Accordingly, ten-year consumption and production capacity data from West Java were analyzed for each commodity over the period 2009–2018 (Statistics of Jawa Barat, 2009, 2010, 2011, 2012, 2013, 2014, 2015, 2016, 2017, 2018). The logistics costs for each region are assumed to be uncertain and calculated based on the price of commodities and diesel fuel price.

In this model, diesel fuel prices are the benchmark for calculating logistics cost because these types of fuel are widely used by mini trucks, which distribute food commodities (Pertamina, 2018b,c,a). Other logistics costs that are calculated in this model are operational cost and cost of investment. Data on the selling price of each commodity was obtained from the Ministry of Trade. The standard for determining the cost of RFH development is based on the Regulation of the Minister of Trade of the Republic of Indonesia Number 37/M-DAG/PER/5/2017 concerning guidelines for the construction and management of Trade facilities.

**Figure 4:**
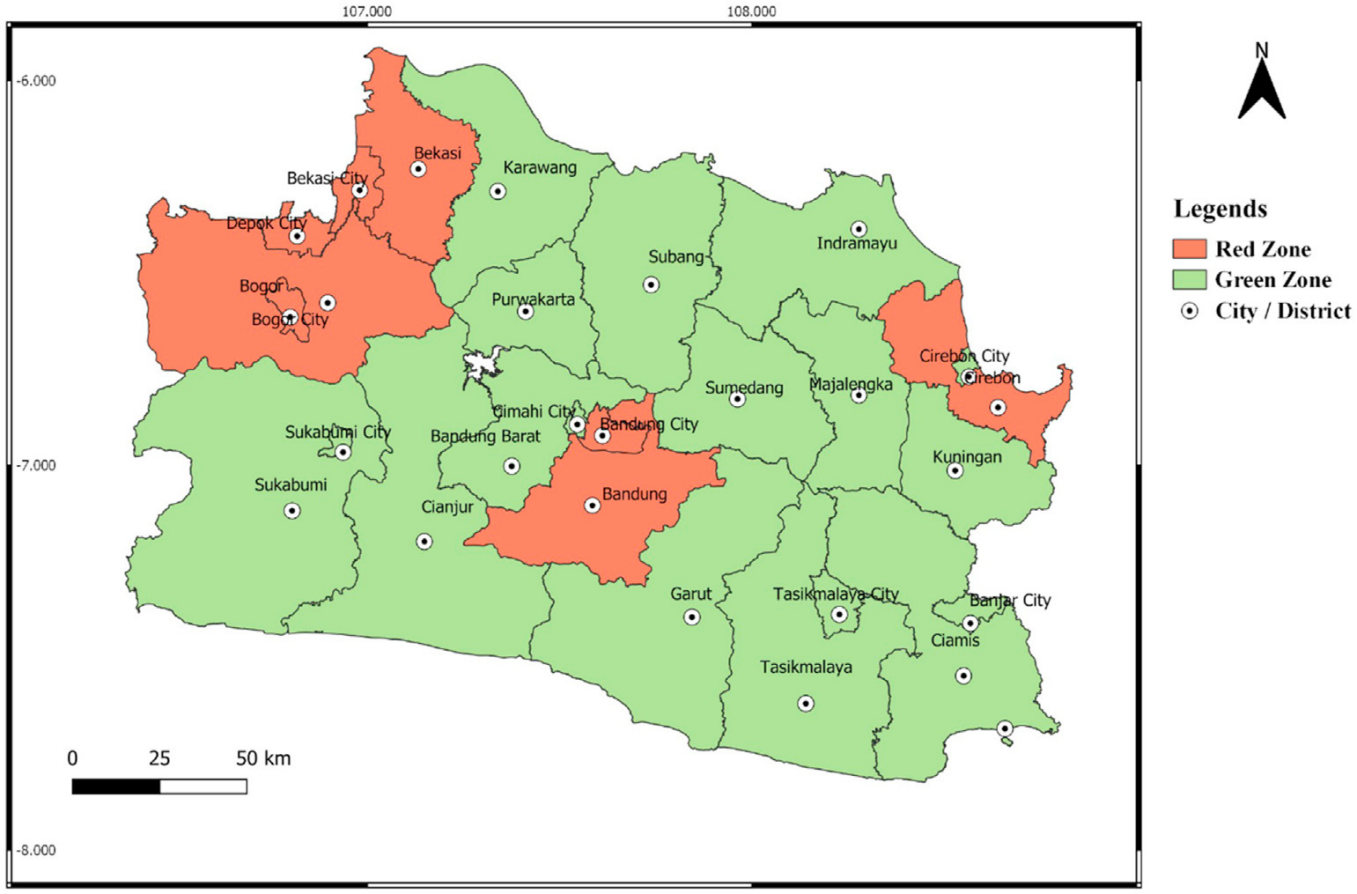
Red zone in West Java.

The RFH location is also determined for the red zones, as presented in Figure 4. As claimed by Pikobar, as of 28 May 2020, 9.04% of patients exposed to COVID-19 were in West Java, placing West Java in the third position for the highest number of COVID-19 cases in Indonesia after DKI Jakarta and East Java. Some red zones in West Java are urban areas with high population density and are far from food supply sources. Moreover, West Java Province does not yet have an RFH to manage food supply sources for the community, and thus faces a severe food security problem. According to Pikobar, 27 districts or cities in West Java Province have patients who tested positive for COVID-19.

In the scenarios constructed, eight districts are considered red zones in Case 1 (based on data from March 21, 2020) and thirteen red zones are identified in Case 2 (based on data on May 20, 2020). All red zones are in urban areas and are the focus of the central and regional governments' monitoring activities. None of the districts in the red zone have a robust food supply network yet, and the Government thus gives high priority to the issue of food security.

In this paper, the RFH model was also designed based on the Governor of West Java's flagship program, "Jabar Juara" (West Java Champion). RFH development in West Java Province emphasizes the concept of the smart supply chain for agricultural development. It aims to provide a stable food supply network and focus on the optimization of local production levels. The West Java Provincial Government also aimed to meet the nutritional needs of the community, reduce food loss and waste, and adopt sustainable agriculture by developing RFHs. Therefore, presidential directives have a significant point of working related to food security (Djalante et al., 2020).

### 4.4 Numerical simulation results

This subsection discusses the results of the numerical simulation for the three scenarios. First, the result for each scenario is discussed, which are then compared at the end of the subsection.

#### 4.4.1 Numerical simulation result: scenario 1

The optimal location for the RFH in scenario 1 is developed based on Case 1 (eight red zones) presented in Table 4, and Case 2 (thirteen red zones), as shown in Table 5. There are eleven alternative RFH locations in Case 1, eight in red zones and three outside. In this scenario, large-scale social distancing is applied as protection aimed to limit space in the red zone. The RFH development area in scenario 1 is at high risk for deficiency of food supply. Further, hunger will continue to increase in the red zone as it cannot depend on the other production areas. The total logistics cost for scenario 1 in Case 1 is Rp 5,212.13/kg production.

**Table 4:** RFH optimal location for case 1 (8 red zones) in scenario 1.

RFHs Location	Red zone (Yes/No)	Capacity (Kilotonne/day)		
		Rice	Chicken Egg	Vegetable
Ciamis	No	2.1201	0.1697	0.5552
Cirebon	Yes	0.5867	0.0394	0.0851
Sukabumi	No	1.9787	0.1328	0.4416
Bogor City	Yes	0.0156	0.0198	0.0058
Depok City	Yes	0.0088	0.0422	0.0070
Bekasi	Yes	0.9790	0.0657	0.0614
Bekasi City	Yes	0.0123	0.0530	0.0119
Bogor	Yes	1.5748	0.1057	0.2305
Bandung	Yes	1.0022	0.0672	0.1508
Bandung City	Yes	0.0331	0.0453	0.0087
Subang	No	2.4948	0.1400	0.3055

**Table 5:** Optimal location of RFH for case 2 (13 red zones) in scenario 1.

RFHs Location	Red zone (Yes/No)	Capacity (Kilotonne/day)		
		Rice	Chicken Egg	Vegetable
Garut	No	1.0528	0.0471	0.1308
Tasikmalaya	Yes	0.4722	0.0317	0.0933
Tasikmalaya City	Yes	0.1787	0.0120	0.0353
Cirebon	Yes	0.5867	0.0394	0.0851
Indramayu	Yes	0.4635	0.0311	0.0915
Majalengka	No	1.9523	0.1210	0.4165
Sukabumi	Yes	0.6634	0.0445	0.1310
Bogor City	Yes	0.0156	0.0198	0.0058
Bekasi	Yes	0.9790	0.0657	0.0614
Bekasi City	Yes	0.0123	0.0530	0.0119
Bogor	No	2.2031	0.1537	0.4351
Cianjur	Yes	0.6095	0.0409	0.1204
Bandung	No	1.7207	0.1430	0.2876
Cimahi City	Yes	0.0086	0.0110	0.0012
Purwakarta	Yes	0.2571	0.0172	0.0508
Karawang	Yes	0.6298	0.0423	0.0366
Pangandaran	Yes	0.1071	0.0072	0.0211

In Case 2, seventeen optimal RFHs must be built, thirteen in the red zone and the rest from other zones. The implementation of large-scale social distancing reduces food distribution from other districts. In scenario 1, there is a change in the amount of RFHs that must be built. As more red zones and locations change, the need for RFH development will continue to increase. It can affect the availability of food in areas affected by large-scale social distancing. The total logistics cost for Scenario 1 in Case 2 is Rp5,212.89/kg.

As discussed before, the greater the number of red zones, the higher is the need for RFH development. This is due to the restriction of the food supply network in the red zones. RFHs should be built in every red zone to maximize demand fulfillment in those areas to avoid an increase in hunger.

In terms of the complexity of the problems, Case 1 in scenario 1 has 4,482 decision variables and 974 parameters, which are involved in 6,522 constraint functions. Meanwhile, Case 2 in scenario 1 has the same decision variables and parameters, which are involved in 6,882 constraint functions. The greater the number of red zones involved in the case, the more complex the model will be.

In this scenario, the rice demand of urban areas that become the epicenter of COVID-19 could not be fulfilled, as presented in Table 6. The area's capacity to produce rice cannot match its total rice needs. The RFHs in four red zones could not import rice from the production areas outside the red zones, due to the policies that forbid distribution from or to the red zones. Thus, more than 8 million citizens in four red zone are in food insecurity.

**Table 6:** Rice fulfillment (Scenario 1).

District	Red zone (Yes/No)		Demand (Kilotonne/day)	Demand Supplied (Kilotonne/day)		Fulfillment Ratio (% fulfilled)	
	Case 1	Case 2		Case 1	Case 2	Case 1	Case 2
Ciamis	No	No	0.3205	0.3205	0.3205	100.00%	100.00 %
Garut	No	No	0.7027	0.7027	0.7027	100.00%	100.00 %
Tasikmalaya	No	Yes	0.4722	0.4722	0.4722	100.00%	100.00 %
Tasikmalaya City	No	Yes	0.1787	0.1787	0.1787	100.00%	100.00 %
Cirebon	Yes	Yes	0.5867	0.5867	0.5867	100.00%	100.00 %
Cirebon City	No	No	0.0853	0.0853	0.0853	100.00%	100.00 %
Indramayu	No	Yes	0.4635	0.4635	0.4635	100.00%	100.00 %
Majalengka	No	No	0.3234	0.3234	0.3234	100.00%	100.00 %
Kuningan	No	No	0.2897	0.2897	0.2897	100.00%	100.00 %
Sukabumi	No	Yes	0.6634	0.6634	0.6634	100.00%	100.00 %
Sukabumi City	No	No	0.0880	0.0880	0.0880	100.00%	100.00 %
Bogor City	Yes	Yes	0.2957	0.0156	0.0156	5.29%	5.29 %
Depok City	Yes	No	0.6283	0.0088	0.6283	1.40%	100.00 %
Bekasi	Yes	Yes	0.9790	0.9790	0.9790	100.00%	100.00 %
Bekasi City	Yes	Yes	0.7905	0.0123	0.0123	1.55%	1.55 %
Bogor	Yes	No	1.5748	1.5748	1.5748	100.00%	100.00 %
Cianjur	No	Yes	0.6095	0.6095	0.6095	100.00%	100.00 %
Bandung	Yes	No	1.0022	1.0022	1.0022	100.00%	100.00 %
Bandung City	Yes	No	0.6750	0.0331	0.6750	4.90%	100.00 %
Cimahi City	No	Yes	0.1639	0.1639	0.0086	100.00%	5.25 %
Sumedang	No	No	0.3100	0.3100	0.3100	100.00%	100.00 %
Bandung Barat	No	No	0.4540	0.4540	0.4540	100.00%	100.00 %
Subang	No	No	0.4257	0.4257	0.4257	100.00%	100.00 %
Purwakarta	No	Yes	0.2571	0.2571	0.2571	100.00%	100.00 %
Karawang	No	Yes	0.6298	0.6298	0.6298	100.00%	100.00 %
Banjar City	No	No	0.0493	0.0493	0.0493	100.00%	100.00 %
Pangandaran	No	Yes	0.1071	0.1071	0.1071	100.00%	100.00 %

Changes in the classification of red zones affect rice demand fulfillment. Depok and Bandung City, whose production capacity cannot fulfill their demand, were classified as red zones in Case 1. However, in Case 2, they are no longer classified as red zones, and so their demand could be fulfilled by other districts, resulting in an increase in the rice fulfillment in those districts. In contrast, Cimahi City was classified as a green zone in Case 1, and other districts could fulfill its demand. However, in Case 2, Cimahi City became a red zone that could not fulfill its demand, resulting in a decrease in rice fulfillment in Cimahi City. Although there are other red-zone status changes in several districts, their demand fulfillment was not affected, e.g., Tasikmalaya, Indramayu, Sukabumi, Bogor, Cianjur, Purwakarta, and Karawang. Their capacity to produce rice can meet their demand, so the demand fulfillment in those districts will not change if they become red zones. Overall, the rice fulfillment increase in Depok and Bandung is higher than the rice fulfillment decrease in Cimahi. At the same time, other changes in red zones did not affect rice demand fulfillment. This makes rice fulfillment in Case 2 is higher than in Case 1.

Unlike rice commodities, demand for chicken eggs can be met by all areas, as presented in Table 7. The 27 districts/cities in West Java can produce chicken eggs without depending on other production areas. The classification of the red zone thus does not affect the fulfillment of chicken egg needs. However, chicken eggs are insufficient to reduce the risk of hunger and the issue of immunity during the COVID-19 pandemic.

**Table 7:** Chicken egg fulfillment (Scenario 1).

District	Red zone (Yes/No)		Demand (Kilotonne/day)	Demand Supplied (Kilotonne/day)		Fulfillment Ratio (% fulfilled)	
	Case 1	Case 2		Case 1	Case 2	Case 1	Case 2
Ciamis	No	No	0.0215	0.0215	0.0215	100.00%	100.00 %
Garut	No	No	0.0471	0.0471	0.0471	100.00%	100.00 %
Tasikmalaya	No	Yes	0.0317	0.0317	0.0317	100.00%	100.00 %
Tasikmalaya City	No	Yes	0.0120	0.0120	0.0120	100.00%	100.00 %
Cirebon	Yes	Yes	0.0394	0.0394	0.0394	100.00%	100.00 %
Cirebon City	No	No	0.0057	0.0057	0.0057	100.00%	100.00 %
Indramayu	No	Yes	0.0311	0.0311	0.0311	100.00%	100.00 %
Majalengka	No	No	0.0217	0.0217	0.0217	100.00%	100.00 %
Kuningan	No	No	0.0194	0.0194	0.0194	100.00%	100.00 %
Sukabumi	No	Yes	0.0445	0.0445	0.0445	100.00%	100.00 %
Sukabumi City	No	No	0.0059	0.0059	0.0059	100.00%	100.00 %
Bogor City	Yes	Yes	0.0198	0.0198	0.0198	100.00%	100.00 %
Depok City	Yes	No	0.0422	0.0422	0.0422	100.00%	100.00 %
Bekasi	Yes	Yes	0.0657	0.0657	0.0657	100.00%	100.00 %
Bekasi City	Yes	Yes	0.0530	0.0530	0.0530	100.00%	100.00 %
Bogor	Yes	No	0.1057	0.1057	0.1057	100.00%	100.00 %
Cianjur	No	Yes	0.0409	0.0409	0.0409	100.00%	100.00 %
Bandung	Yes	No	0.0672	0.0672	0.0672	100.00%	100.00 %
Bandung City	Yes	No	0.0453	0.0453	0.0453	100.00%	100.00 %
Cimahi City	No	Yes	0.0110	0.0110	0.0110	100.00%	100.00 %
Sumedang	No	No	0.0208	0.0208	0.0208	100.00%	100.00 %
Bandung Barat	No	No	0.0305	0.0305	0.0305	100.00%	100.00 %
Subang	No	No	0.0286	0.0286	0.0286	100.00%	100.00 %
Purwakarta	No	Yes	0.0172	0.0172	0.0172	100.00%	100.00 %
Karawang	No	Yes	0.0423	0.0423	0.0423	100.00%	100.00 %
Banjar City	No	No	0.0033	0.0033	0.0033	100.00%	100.00 %
Pangandaran	No	Yes	0.0072	0.0072	0.0072	100.00%	100.00 %

Changes in the classification of red zones did not affect the fulfillment of the egg demand. When green zones in Case 1 become red zones in Case 2, their egg demand could not be supported by other districts. However, their own capacity to produce eggs could fulfill their demand, and so the demand fulfillment in those districts could be maintained. When a green zone in Case 1 became a red zone in Case 2, the eggs produced there could no longer be distributed to other districts. However, other districts produced sufficient eggs, and so the egg demand in green zones was still fulfilled, thus maintaining the overall fulfillment of egg demand. When the red zone in Case 1 became a green zone in Case 2, the egg produced could be distributed to another green zone. Further, their demand could be fulfilled by another green zone, which makes for a better and more productive food supply network.

About eight districts/cities in Case 1 and nine red zones in Case 2 could not meet vegetable demand, as presented in Table 8. This was because these zones are not the main vegetable-producing area. The main production areas were restricted from distributing the vegetable yields to the red zones, which makes the vegetable fulfillment in this scenario even worse. Based on the rate of vegetable consumption in each district, it is estimated that more than 13 million people in West Java could not fulfill their vegetable needs.

**Table 8:** Vegetable fulfillment (Scenario 1).

District	Red zone (Yes/No)		Demand (Kilotonne/day)	Demand Supplied (Kilotonne/day)		Fulfillment Ratio (% fulfilled)	
	Case 1	Case 2		Case 1	Case 2	Case 1	Case 2
Ciamis	No	No	0.0633	0.0633	-	100.00%	0.00 %
Garut	No	No	0.1388	0.1388	0.1308	100.00%	94.23 %
Tasikmalaya	No	Yes	0.0933	0.0933	0.0933	100.00%	100.00 %
Tasikmalaya City	No	Yes	0.0353	0.0353	0.0353	100.00%	100.00 %
Cirebon	Yes	Yes	0.1159	0.0851	0.0851	73.43%	73.43 %
Cirebon City	No	No	0.0168	0.0168	0.0168	100.00%	100.00 %
Indramayu	No	Yes	0.0915	0.0915	0.0915	100.00%	100.00 %
Majalengka	No	No	0.0639	0.0639	0.0639	100.00%	100.00 %
Kuningan	No	No	0.0572	0.0572	0.0572	100.00%	100.00 %
Sukabumi	No	Yes	0.1310	0.1310	0.1310	100.00%	100.00 %
Sukabumi City	No	No	0.0174	0.0174	-	100.00%	0.00 %
Bogor City	Yes	Yes	0.0584	0.0058	0.0058	9.95%	9.95 %
Depok City	Yes	No	0.1241	0.0070	0.1241	5.62%	100.00 %
Bekasi	Yes	Yes	0.1933	0.0614	0.0614	31.75%	31.75 %
Bekasi City	Yes	Yes	0.1561	0.0119	0.0119	7.64%	7.64 %
Bogor	Yes	No	0.3110	0.2305	0.3110	74.11%	100.00 %
Cianjur	No	Yes	0.1204	0.1204	0.1204	100.00%	100.00 %
Bandung	Yes	No	0.1979	0.1508	0.1979	76.19%	100.00 %
Bandung City	Yes	No	0.1333	0.0087	0.1333	6.52%	100.00 %
Cimahi City	No	Yes	0.0324	0.0324	0.0012	100.00%	3.58 %
Sumedang	No	No	0.0612	0.0612	0.0612	100.00%	100.00 %
Bandung Barat	No	No	0.0897	0.0897	0.0897	100.00%	100.00 %
Subang	No	No	0.0841	0.0841	0.0841	100.00%	100.00 %
Purwakarta	No	Yes	0.0508	0.0508	0.0508	100.00%	100.00 %
Karawang	No	Yes	0.1244	0.1244	0.0366	100.00%	29.42 %
Banjar City	No	No	0.0097	0.0097	-	100.00%	0.00 %
Pangandaran	No	Yes	0.0211	0.0211	0.0211	100.00%	100.00 %

Changes in the classification of red zone affect vegetable fulfillment. The fulfillment ratio of a red zone in Case 1 that changed into a green zone in Case 2 will remain the same or increase. Whenever a red zone becomes a green zone, its demand can be fulfilled by other districts, and so its demand fulfillment increased or at least remains the same. In contrast, the fulfillment ratio of a green zone in Case 1 that changed in a red zone in Case 2 will remain the same or decrease. Whenever a green zone becomes a red zone, its demand cannot be fulfilled by other districts anymore, and so their demand fulfillment decreases or at least remains the same. The green zones in Case 1 that changed into red zones in Case 2 are predominantly in suburban areas, with sufficient production capacity to fulfill their own demand. The red zones in Case 1 that changed into the green zone in Case 2 are predominantly urban, with a high population density, resulting in more people requiring vegetable supply. This makes the vegetable fulfillment in Case 2 higher than in Case 1.

Scenario 1 restricted the food supply network from the production area to the RFHs in the red zones, who are then forced to produce food. The scenario creates a significant RFH development cost because every time an additional red zone comes up, the government must build an RFH. Limited movement causes shortage of some food commodities. This scenario cannot optimize the RFH function as an aggregator between food production and consumer areas. These conditions result in food insecurity and malnutrition.

**Figure 5:**
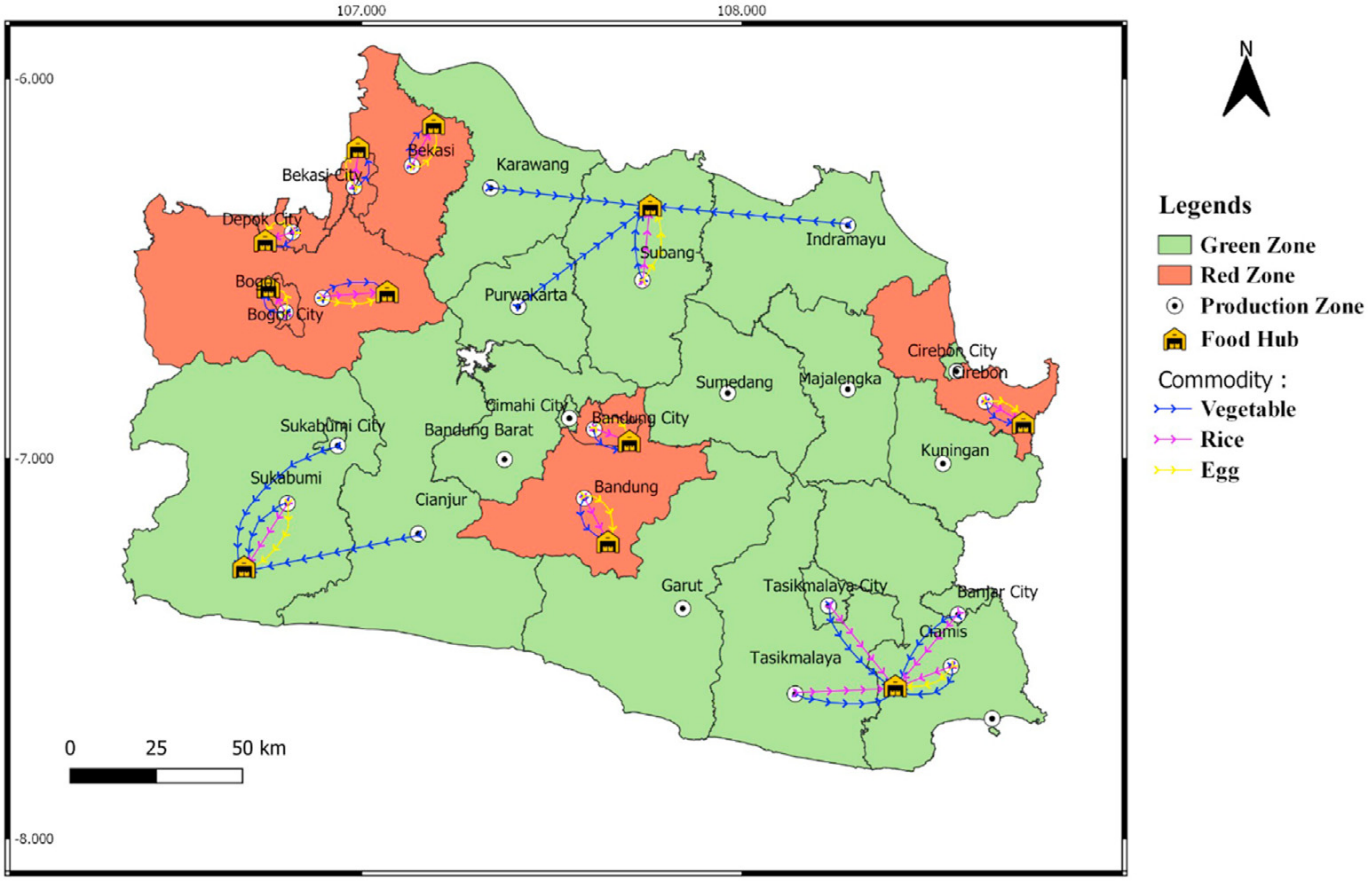
Food supply network from production zone to RFH for case 1 in scenario 1.

**Figure 6:**
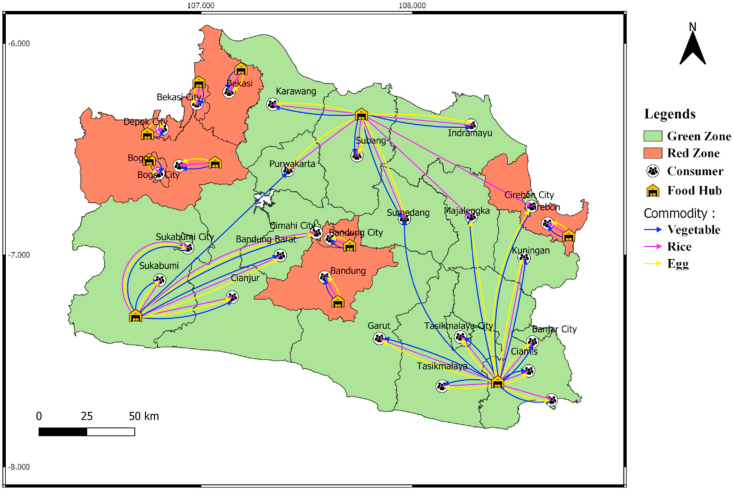
Food supply network from RFH to consumer for case 1 in scenario 1.

Scenario 1 is not compatible when applied in the long term. It would cause certain areas to be isolated from production areas and reduce the fulfillment of food demand. Although RFHs are required to serve all consumers, the supply does not match the consumption needs. In Figures 5 and 6, the RFH is not optimistic about providing food for the community.

#### 4.4.2 Numerical simulation result: scenario 2

The optimal location for the RFH in scenario 2 is developed based on Case 1 (eight red zones) in Table 9, and Case 2 (thirteen red zones) in Table 10. Scenario 2 was built, in which partial social distancing conditions were applied in the red zone areas. Although the production center can supply food to the RFHs in the red zone, the addition of the red zone still affects the number of RFHs that must be built. The total RFHs built is eleven in Case 1, eight in the red zones, and the rest outside. The total logistics cost for scenario 2 in Case 1 is Rp 5,211.35/kg, and the fulfillment ratio of rice and chicken egg reaches 100%; only the demand for vegetables could not be fulfilled.

**Table 9:** RFH optimal location for case 1 (8 red zones) in scenario 2.

RFHs Location	Red Zone (Yes/No)	Capacity (Kilotonne/day)		
		Rice	Chicken Egg	Vegetable
Ciamis	No	2.1201	0.1697	0.6215
Cirebon	Yes	0.5867	0.0394	0.1159
Sukabumi	No	1.9787	0.1328	0.1792
Bogor City	Yes	0.2957	0.0198	0.0584
Depok City	Yes	0.6283	0.0422	0.1241
Bekasi	Yes	0.9790	0.0657	0.1933
Bekasi City	Yes	0.7905	0.0530	0.1561
Bogor	Yes	1.5748	0.1057	0.3110
Bandung	Yes	1.0022	0.0672	0.1979
Bandung City	Yes	0.6750	0.0453	0.1333
Subang	No	2.4948	0.1400	0.3017

**Table 10:** Optimal RFH location for case 2 (13 red zones) in scenario 2.

RFHs Location	Red Zone (Yes/No)	Capacity (Kilotonne/day)		
		Rice	Chicken Egg	Vegetable
Tasikmalaya	Yes	0.4722	0.0317	0.0933
Tasikmalaya City	Yes	0.1787	0.0120	0.0353
Cirebon	Yes	0.5867	0.0394	0.1159
Indramayu	Yes	0.4635	0.0311	0.0915
Sukabumi	Yes	0.6634	0.0445	0.1310
Bogor City	Yes	0.2957	0.0198	0.0058
Bekasi	Yes	0.9790	0.0657	0.1933
Bekasi City	Yes	0.7905	0.0530	0.1561
Bogor	No	2.2031	0.1537	0.1399
Cianjur	Yes	0.6095	0.0409	0.1204
Bandung	No	1.4852	0.1430	0.2671
Cimahi City	Yes	0.1639	0.0110	0.0324
Sumedang	No	3.2406	0.1682	0.4370
Purwakarta	Yes	0.2571	0.0172	0.0508
Karawang	Yes	0.6298	0.0423	0.1244
Pangandaran	Yes	0.1071	0.0072	0.0211

If the vegetables produced in West Java is sufficient to fulfill the overall demand, RFH development will not be affected by the changes in the classification of red zones. However, since this is not the case, RFHs should be built in the red zones so that the vegetables produced in the red zones could be distributed to fulfill their demand. This condition makes the number of RFHs increase in Case 2, in which sixteen RFHs are built. The total logistic cost for scenario 2 in Case 2 is Rp5,214.46/Kg.

In terms of the complexity of the problems, Case 1 in scenario 2 has 4,498 decision variables and 975 parameters, which are involved in 5,658 constraint functions. Meanwhile, Case 2 in scenario 1 has 4,508 decision variables and 975 parameters involved in 5,868 constraint functions. The greater the number of red zones involved in the case, the more complex the model will be.

In this scenario, RFHs become crucial to maximize the supply of products. According to Table 11, all areas in Cases 1 and 2 could fulfill rice demand, as rice production in every area in West Java is sufficient to satisfy their demand. The application of partial social distancing helps RFHs to provide for the rice needs in each RFH. Further, hygiene and food safety standards must be implemented in the case of partial social distancing in food distribution.

**Table 11:** Rice fulfillment (Scenario 2).

District	Red zone (Yes/No)		Demand (Kilotonne/day)	Demand Supplied (Kilotonne/day)		Fulfillment Ratio (% fulfilled)	
	Case 1	Case 2		Case 1	Case 2	Case 1	Case 2
Ciamis	No	No	0.3205	0.3205	0.3205	100.00%	100.00 %
Garut	No	No	0.7027	0.7027	0.7027	100.00%	100.00 %
Tasikmalaya	No	Yes	0.4722	0.4722	0.4722	100.00%	100.00 %
Tasikmalaya City	No	Yes	0.1787	0.1787	0.1787	100.00%	100.00 %
Cirebon	Yes	Yes	0.5867	0.5867	0.5867	100.00%	100.00 %
Cirebon City	No	No	0.0853	0.0853	0.0853	100.00%	100.00 %
Indramayu	No	Yes	0.4635	0.4635	0.4635	100.00%	100.00 %
Majalengka	No	No	0.3234	0.3234	0.3234	100.00%	100.00 %
Kuningan	No	No	0.2897	0.2897	0.2897	100.00%	100.00 %
Sukabumi	No	Yes	0.6634	0.6634	0.6634	100.00%	100.00 %
Sukabumi City	No	No	0.0880	0.0880	0.0880	100.00%	100.00 %
Bogor City	Yes	Yes	0.2957	0.2957	0.2957	100.00%	100.00 %
Depok City	Yes	No	0.6283	0.6283	0.6283	100.00%	100.00 %
Bekasi	Yes	Yes	0.9790	0.9790	0.9790	100.00%	100.00 %
Bekasi City	Yes	Yes	0.7905	0.7905	0.7905	100.00%	100.00 %
Bogor	Yes	No	1.5748	1.5748	1.5748	100.00%	100.00 %
Cianjur	No	Yes	0.6095	0.6095	0.6095	100.00%	100.00 %
Bandung	Yes	No	1.0022	1.0022	1.0022	100.00%	100.00 %
Bandung City	Yes	No	0.6750	0.6750	0.6750	100.00%	100.00 %
Cimahi City	No	Yes	0.1639	0.1639	0.1639	100.00%	100.00 %
Sumedang	No	No	0.3100	0.3100	0.3100	100.00%	100.00 %
Bandung Barat	No	No	0.4540	0.4540	0.4540	100.00%	100.00 %
Subang	No	No	0.4257	0.4257	0.4257	100.00%	100.00 %
Purwakarta	No	Yes	0.2571	0.2571	0.2571	100.00%	100.00 %
Karawang	No	Yes	0.6298	0.6298	0.6298	100.00%	100.00 %
Banjar City	No	No	0.0493	0.0493	0.0493	100.00%	100.00 %
Pangandaran	No	Yes	0.1071	0.1071	0.1071	100.00%	100.00 %

The changes in the classification of red zones did not affect the rice demand fulfillment in scenario 2. This is because when a green zone in Case 1 became red zone in Case 2, their produced rice could no longer be distributed to other districts but there other districts produced sufficient rice, which maintains the fulfillment of rice demand. When a red zone in Case 1 became a green zone in Case 2, fulfillment of rice demand improved because the rice produced in those districts is no longer restricted, which provides additional rice supplies from those districts, resulting in a the better and more effective food supply network.

The chicken egg requirement in scenario 2 can be fulfilled 100% in each district of West Java Province, as presented in Table 12. The partial social distancing for some commodities could build customer confidence to reduce risk exposure and guarantee universal health coverage. These results carry obvious implications for household food security and nutrition, particularly for those in the red zone.

**Table 12:** Chicken egg fulfillment (Scenario 2).

District	Red zone (Yes/No)		Demand (Kilotonne/day)	Demand Supplied (Kilotonne/day)		Fulfillment Ratio (% fulfilled)	
	Case 1	Case 2		Case 1	Case 2	Case 1	Case 2
Ciamis	No	No	0.0215	0.0215	0.0215	100.00%	100.00 %
Garut	No	No	0.0471	0.0471	0.0471	100.00%	100.00 %
Tasikmalaya	No	Yes	0.0317	0.0317	0.0317	100.00%	100.00 %
Tasikmalaya City	No	Yes	0.0120	0.0120	0.0120	100.00%	100.00 %
Cirebon	Yes	Yes	0.0394	0.0394	0.0394	100.00%	100.00 %
Cirebon City	No	No	0.0057	0.0057	0.0057	100.00%	100.00 %
Indramayu	No	Yes	0.0311	0.0311	0.0311	100.00%	100.00 %
Majalengka	No	No	0.0217	0.0217	0.0217	100.00%	100.00 %
Kuningan	No	No	0.0194	0.0194	0.0194	100.00%	100.00 %
Sukabumi	No	Yes	0.0445	0.0445	0.0445	100.00%	100.00 %
Sukabumi City	No	No	0.0059	0.0059	0.0059	100.00%	100.00 %
Bogor City	Yes	Yes	0.0198	0.0198	0.0198	100.00%	100.00 %
Depok City	Yes	No	0.0422	0.0422	0.0422	100.00%	100.00 %
Bekasi	Yes	Yes	0.0657	0.0657	0.0657	100.00%	100.00 %
Bekasi City	Yes	Yes	0.0530	0.0530	0.0530	100.00%	100.00 %
Bogor	Yes	No	0.1057	0.1057	0.1057	100.00%	100.00 %
Cianjur	No	Yes	0.0409	0.0409	0.0409	100.00%	100.00 %
Bandung	Yes	No	0.0672	0.0672	0.0672	100.00%	100.00 %
Bandung City	Yes	No	0.0453	0.0453	0.0453	100.00%	100.00 %
Cimahi City	No	Yes	0.0110	0.0110	0.0110	100.00%	100.00 %
Sumedang	No	No	0.0208	0.0208	0.0208	100.00%	100.00 %
Bandung Barat	No	No	0.0305	0.0305	0.0305	100.00%	100.00 %
Subang	No	No	0.0286	0.0286	0.0286	100.00%	100.00 %
Purwakarta	No	Yes	0.0172	0.0172	0.0172	100.00%	100.00 %
Karawang	No	Yes	0.0423	0.0423	0.0423	100.00%	100.00 %
Banjar City	No	No	0.0033	0.0033	0.0033	100.00%	100.00 %
Pangandaran	No	Yes	0.0072	0.0072	0.0072	100.00%	100.00 %

The changes in the classification of red zones did not affect the egg demand fulfillment in scenario 2. This is because when the green zone in Case 1 becomes a red zone in Case 2, the egg produced there could no longer be distributed to other districts. However, other districts could produce sufficient eggs, which helps maintain the fulfillment of egg demand. When the red zone in Case 1 became a green zone in Case 2, the egg demand fulfillment improved because the eggs produced in those districts is no longer restricted, which increases egg supply from those districts.

In this scenario, partial social distancing cannot meet the needs of vegetable consumption, as presented in Table 13. Therefore, vegetable needs can be substituted with other vegetable commodities to meet daily food fiber needs. There are about 3.7 million people in West Java whose vegetable needs would not be fulfilled if there is no substitute in the form of other vegetable commodities.

**Table 13:** Vegetable fulfillment (Scenario 2).

District	Red zone (Yes/No)		Demand (Kilotonne/day)	Demand Supplied (Kilotonne/day)		Fulfillment Ratio (% fulfilled)	
	Case 1	Case 2		Case 1	Case 2	Case 1	Case 2
Ciamis	No	No	0.0633	0.0633	-	100.00%	0.00 %
Garut	No	No	0.1388	0.0874	-	62.97%	0.00 %
Tasikmalaya	No	Yes	0.0933	0.0933	0.0933	100.00%	100.00 %
Tasikmalaya City	No	Yes	0.0353	0.0353	0.0353	100.00%	100.00 %
Cirebon	Yes	Yes	0.1159	0.1159	0.1159	100.00%	100.00 %
Cirebon City	No	No	0.0168	0.0168	0.0168	100.00%	100.00 %
Indramayu	No	Yes	0.0915	0.0915	0.0915	100.00%	100.00 %
Majalengka	No	No	0.0639	0.0639	0.0639	100.00%	100.00 %
Kuningan	No	No	0.0572	0.0572	0.0572	100.00%	100.00 %
Sukabumi	No	Yes	0.1310	-	0.1310	0.00%	100.00 %
Sukabumi City	No	No	0.0174	-	-	0.00%	0.00 %
Bogor City	Yes	Yes	0.0584	0.0584	0.0058	100.00%	9.95 %
Depok City	Yes	No	0.1241	0.1241	0.1241	100.00%	100.00 %
Bekasi	Yes	Yes	0.1933	0.1933	0.1933	100.00%	100.00 %
Bekasi City	Yes	Yes	0.1561	0.1561	0.1561	100.00%	100.00 %
Bogor	Yes	No	0.3110	0.3110	0.0159	100.00%	5.10 %
Cianjur	No	Yes	0.1204	0.1204	0.1204	100.00%	100.00 %
Bandung	Yes	No	0.1979	0.1979	0.1979	100.00%	100.00 %
Bandung City	Yes	No	0.1333	0.1333	0.1333	100.00%	100.00 %
Cimahi City	No	Yes	0.0324	0.0324	0.0324	100.00%	100.00 %
Sumedang	No	No	0.0612	0.0612	0.0612	100.00%	100.00 %
Bandung Barat	No	No	0.0897	0.0897	0.0897	100.00%	100.00 %
Subang	No	No	0.0841	0.0841	0.0841	100.00%	100.00 %
Purwakarta	No	Yes	0.0508	0.0508	0.0508	100.00%	100.00 %
Karawang	No	Yes	0.1244	0.1244	0.1244	100.00%	100.00 %
Banjar City	No	No	0.0097	0.0097	-	100.00%	0.00 %
Pangandaran	No	Yes	0.0211	0.0211	0.0211	100.00%	100.00 %

Changes in the classification of red zones affect vegetable demand fulfillment. This is because the vegetables produced in West Java are less than the demand for them. When the red zones in Case 1 become green zones in Case 2, the vegetable produced there could be distributed to other districts, which strengthens the food supply network. However, when a green zone in Case 1 becomes a red zone in Case 2, the produced vegetable could no longer be distributed to other districts anymore. Meanwhile, the vegetable produced in other districts is insufficient to fulfill the overall demand. Thus, the greater the number of red zones, the lower the vegetable supply, which results in a decrease in vegetable fulfillment. This condition could worsen if the green zones that change into red zones are predominantly in suburban areas with high vegetable production capacity.

In Case 1, the vegetable fulfillment reached maximum with all the vegetable produced. All vegetable produced are distributed in Case 1. However, as the number of red zones increased in Case 2, vegetable fulfillment decreased. Cianjur is one of the suburban areas with high vegetable production capacity. When Cianjur becomes a red zone in Case 2, West Java loses one of its main vegetable production sources, which makes the vegetable fulfillment in Case 2 lower than Case in 1.

**Figure 7:**
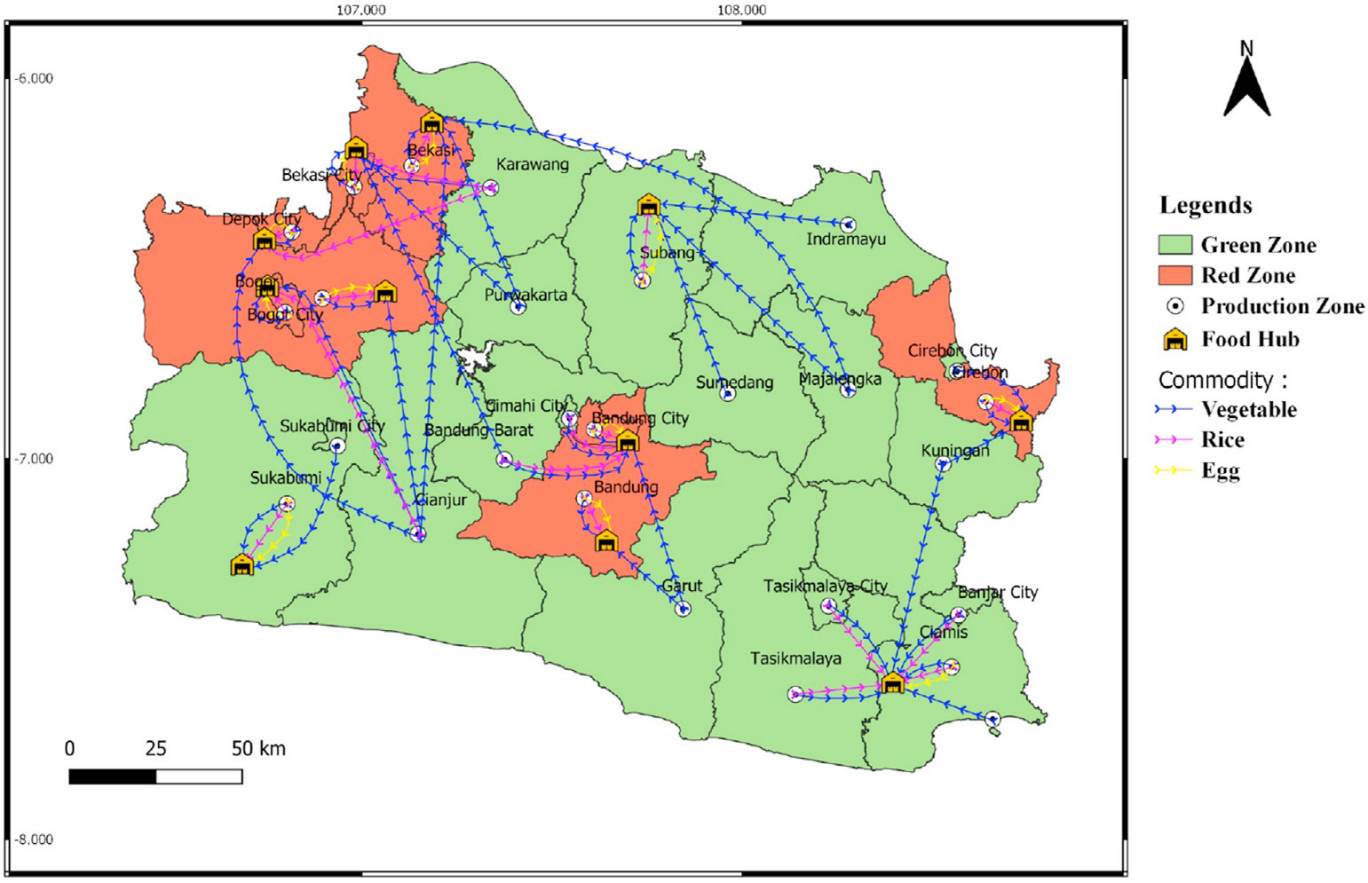
Food supply network from production zone to RFH for case 1 in scenario 2.

**Figure 8:**
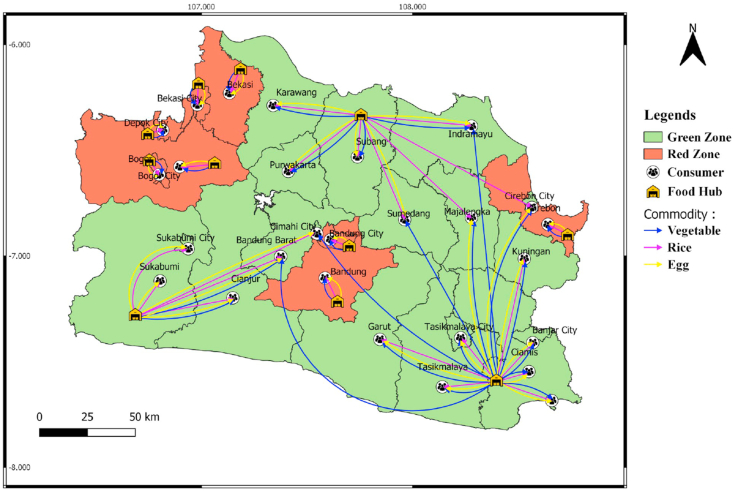
Food supply network from RFH to consumer for case 1 in scenario 2.

Although RFHs can receive supplies from several nearby production areas, the red zones are restricted from supplying food to other districts, as shown in Figures 7 and 8. This distribution model is still safe if the area affected by the pandemic is an urban area. However, if the pandemic extends to areas of food production centers in West Java, food shortages is likely to occur in various districts.

The food supply network of RFHs can only serve consumers who are in the red zone. This scenario is safe if the red zones are confined to urban areas—this model is designed to ensure that customers in the red zone have food security. However, the large number of RFHs has caused high RFH development costs.

#### 4.4.3 Numerical simulation result: scenario 3

The optimal location for the RFH in scenario 3 is developed based on Case 1 (eight red zones) in Table 14, and Case 2 (thirteen red zones) is shown in Table 15. Unlike scenarios 1 and 2, in scenario 3, RFH development is not affected by the change in the classification and increase of red zones. The number of RFH that must be built does not change in scenario 3, which has implications for the cheaper RFH development. The RFH is built near central production areas, and can optimized to supply food to a broad range of consumers. The total logistics cost in model 3 is thus lower than in scenario 1 and 2, at Rp 5,208.38/Kg. The demand fulfillment of all food commodities reaches its maximum in this scenario. The number of RFHs built in this scenario is less than in scenarios 1 and 2, whereas, the total capacity of all RFHs is higher. The food supply network in this scenario is more effective than in scenarios 1 and 2.

**Table 14:** Optimal location of RFH for case 1 (8 red zones) in scenario 3.

RFHs Location	Red Zone (Yes/No)	Capacity (Kilotonne/day)		
		Rice	Chicken Egg	Vegetable
Ciamis	No	1.9088	0.1228	0.5988
Cirebon	Yes	1.7486	0.1381	0.3179
Bogor	Yes	1.7136	0.1676	0.4344
Cianjur	No	2.5784	0.0916	0.3219
Bandung	Yes	1.4852	0.1537	0.3049
Karawang	No	3.6914	0.2068	0.4146

**Table 15:** Optimal location of RFH for case 2 (13 red zones) in scenario 3.

RFHs Location	Red Zone (Yes/No)	Capacity (Kilotonne/day)		
		Rice	Chicken Egg	Vegetable
Ciamis	No	1.9088	0.1228	0.5988
Cirebon	Yes	1.7486	0.1381	0.3179
Bogor	No	1.7136	0.1676	0.4344
Cianjur	Yes	2.5784	0.0913	0.3212
Bandung	No	1.4852	0.1540	0.3049
Karawang	Yes	3.6914	0.2068	0.4146

In terms of the complexity of the problem, Case 1 in scenario 3 has 4,514 decision variables and 975 parameters, which are involved in 4,746 constraint functions. Meanwhile, Case 2 in scenario 1 has 4,524 decision variables and 975 parameters, which are involved in 4,776 constraint functions. The greater the number of red zones involved in the case, the more complex the model will be.

In scenario 3, the RFH development can optimize food security for the whole community. The food supply network that carries out normal conditions could impact the economic circulation. Food security in the COVID-19 pandemic is vital for the survival and health of society. For the people of West Java, rice supplies are crucial, especially when there is social distancing. The needs of rice in West Java can be fulfilled, as presented in Table 16.

**Table 16:** Rice fulfillment (Scenario 3).

District	Red zone (Yes/No)		Demand (Kilotonne/day)	Demand Supplied (Kilotonne/day)		Fulfillment Ratio (% fulfilled)	
	Case 1	Case 2		Case 1	Case 2	Case 1	Case 2
Ciamis	No	No	0.3205	0.3205	0.3205	100.00%	100.00 %
Garut	No	No	0.7027	0.7027	0.7027	100.00%	100.00 %
Tasikmalaya	No	Yes	0.4722	0.4722	0.4722	100.00%	100.00 %
Tasikmalaya City	No	Yes	0.1787	0.1787	0.1787	100.00%	100.00 %
Cirebon	Yes	Yes	0.5867	0.5867	0.5867	100.00%	100.00 %
Cirebon City	No	No	0.0853	0.0853	0.0853	100.00%	100.00 %
Indramayu	No	Yes	0.4635	0.4635	0.4635	100.00%	100.00 %
Majalengka	No	No	0.3234	0.3234	0.3234	100.00%	100.00 %
Kuningan	No	No	0.2897	0.2897	0.2897	100.00%	100.00 %
Sukabumi	No	Yes	0.6634	0.6634	0.6634	100.00%	100.00 %
Sukabumi City	No	No	0.0880	0.0880	0.0880	100.00%	100.00 %
Bogor City	Yes	Yes	0.2957	0.2957	0.2957	100.00%	100.00 %
Depok City	Yes	No	0.6283	0.6283	0.6283	100.00%	100.00 %
Bekasi	Yes	Yes	0.9790	0.9790	0.9790	100.00%	100.00 %
Bekasi City	Yes	Yes	0.7905	0.7905	0.7905	100.00%	100.00 %
Bogor	Yes	No	1.5748	1.5748	1.5748	100.00%	100.00 %
Cianjur	No	Yes	0.6095	0.6095	0.6095	100.00%	100.00 %
Bandung	Yes	No	1.0022	1.0022	1.0022	100.00%	100.00 %
Bandung City	Yes	No	0.6750	0.6750	0.6750	100.00%	100.00 %
Cimahi City	No	Yes	0.1639	0.1639	0.1639	100.00%	100.00 %
Sumedang	No	No	0.3100	0.3100	0.3100	100.00%	100.00 %
Bandung Barat	No	No	0.4540	0.4540	0.4540	100.00%	100.00 %
Subang	No	No	0.4257	0.4257	0.4257	100.00%	100.00 %
Purwakarta	No	Yes	0.2571	0.2571	0.2571	100.00%	100.00 %
Karawang	No	Yes	0.6298	0.6298	0.6298	100.00%	100.00 %
Banjar City	No	No	0.0493	0.0493	0.0493	100.00%	100.00 %
Pangandaran	No	Yes	0.1071	0.1071	0.1071	100.00%	100.00 %

The needs of the chicken egg in West Java Province can also be fulfilled, as presented in Table 17. Six districts control up to 43.72% of the total production of chicken eggs in West Java Province. This scenario can guarantee the sustainability and availability of egg supply in each customer area in West Java Province.

**Table 17:** Chicken egg fulfillment (Scenario 3).

District	Red zone (Yes/No)		Demand (Kilotonne/day)	Demand Supplied (Kilotonne/day)		Fulfillment Ratio (% fulfilled)	
	Case 1	Case 2		Case 1	Case 2	Case 1	Case 2
Ciamis	No	No	0.0215	0.0215	0.0215	100.00%	100.00 %
Garut	No	No	0.0471	0.0471	0.0471	100.00%	100.00 %
Tasikmalaya	No	Yes	0.0317	0.0317	0.0317	100.00%	100.00 %
Tasikmalaya City	No	Yes	0.0120	0.0120	0.0120	100.00%	100.00 %
Cirebon	Yes	Yes	0.0394	0.0394	0.0394	100.00%	100.00 %
Cirebon City	No	No	0.0057	0.0057	0.0057	100.00%	100.00 %
Indramayu	No	Yes	0.0311	0.0311	0.0311	100.00%	100.00 %
Majalengka	No	No	0.0217	0.0217	0.0217	100.00%	100.00 %
Kuningan	No	No	0.0194	0.0194	0.0194	100.00%	100.00 %
Sukabumi	No	Yes	0.0445	0.0445	0.0445	100.00%	100.00 %
Sukabumi City	No	No	0.0059	0.0059	0.0059	100.00%	100.00 %
Bogor City	Yes	Yes	0.0198	0.0198	0.0198	100.00%	100.00 %
Depok City	Yes	No	0.0422	0.0422	0.0422	100.00%	100.00 %
Bekasi	Yes	Yes	0.0657	0.0657	0.0657	100.00%	100.00 %
Bekasi City	Yes	Yes	0.0530	0.0530	0.0530	100.00%	100.00 %
Bogor	Yes	No	0.1057	0.1057	0.1057	100.00%	100.00 %
Cianjur	No	Yes	0.0409	0.0409	0.0409	100.00%	100.00 %
Bandung	Yes	No	0.0672	0.0672	0.0672	100.00%	100.00 %
Bandung City	Yes	No	0.0453	0.0453	0.0453	100.00%	100.00 %
Cimahi City	No	Yes	0.0110	0.0110	0.0110	100.00%	100.00 %
Sumedang	No	No	0.0208	0.0208	0.0208	100.00%	100.00 %
Bandung Barat	No	No	0.0305	0.0305	0.0305	100.00%	100.00 %
Subang	No	No	0.0286	0.0286	0.0286	100.00%	100.00 %
Purwakarta	No	Yes	0.0172	0.0172	0.0172	100.00%	100.00 %
Karawang	No	Yes	0.0423	0.0423	0.0423	100.00%	100.00 %
Banjar City	No	No	0.0033	0.0033	0.0033	100.00%	100.00 %
Pangandaran	No	Yes	0.0072	0.0072	0.0072	100.00%	100.00 %

Two cities could not meet vegetable demand, and one district cannot fulfill 100% of the vegetable needs in the area, as presented in Table 18. Around 3.7 million people are threatened with a shortage of vegetable supplies. As stated earlier, the lack of fulfillment of vegetables can be substituted with other commodities that are not considered in this model. Therefore, vegetable consumption fulfillment can be reconsidered by adding consumption and production components of other vegetable commodities produced locally.

**Table 18:** Vegetable fulfillment (Scenario 3).

District	Red zone (Yes/No)		Demand (Kilotonne/day)	Demand Supplied (Kilotonne/day)		Fulfillment Ratio (% fulfilled)	
	Case 1	Case 2		Case 1	Case 2	Case 1	Case 2
Ciamis	No	No	0.0633	0.0633	0.0633	100.00%	100.00 %
Garut	No	No	0.1388	0.0874	0.0874	62.97%	62.97 %
Tasikmalaya	No	Yes	0.0933	0.0933	0.0933	100.00%	100.00 %
Tasikmalaya City	No	Yes	0.0353	0.0353	0.0353	100.00%	100.00 %
Cirebon	Yes	Yes	0.1159	0.1159	0.1159	100.00%	100.00 %
Cirebon City	No	No	0.0168	0.0168	0.0168	100.00%	100.00 %
Indramayu	No	Yes	0.0915	0.0915	0.0915	100.00%	100.00 %
Majalengka	No	No	0.0639	0.0639	0.0639	100.00%	100.00 %
Kuningan	No	No	0.0572	0.0572	0.0572	100.00%	100.00 %
Sukabumi	No	Yes	0.1310	-	-	0.00%	0.00 %
Sukabumi City	No	No	0.0174	-	-	0.00%	0.00 %
Bogor City	Yes	Yes	0.0584	0.0584	0.0584	100.00%	100.00 %
Depok City	Yes	No	0.1241	0.1233	0.1233	99.41%	99.41 %
Bekasi	Yes	Yes	0.1933	0.1933	0.1933	100.00%	100.00 %
Bekasi City	Yes	Yes	0.1561	0.1561	0.1561	100.00%	100.00 %
Bogor	Yes	No	0.3110	0.3110	0.3110	100.00%	100.00 %
Cianjur	No	Yes	0.1204	0.1204	0.1204	100.00%	100.00 %
Bandung	Yes	No	0.1979	0.1979	0.1979	100.00%	100.00 %
Bandung City	Yes	No	0.1333	0.1333	0.1333	100.00%	100.00 %
Cimahi City	No	Yes	0.0324	0.0324	0.0324	100.00%	100.00 %
Sumedang	No	No	0.0612	0.0612	0.0612	100.00%	100.00 %
Bandung Barat	No	No	0.0897	0.0897	0.0897	100.00%	100.00 %
Subang	No	No	0.0841	0.0841	0.0841	100.00%	100.00 %
Purwakarta	No	Yes	0.0508	0.0508	0.0508	100.00%	100.00 %
Karawang	No	Yes	0.1244	0.1244	0.1244	100.00%	100.00 %
Banjar City	No	No	0.0097	0.0097	0.0097	100.00%	100.00 %
Pangandaran	No	Yes	0.0211	0.0211	0.0211	100.00%	100.00 %

**Figure 9:**
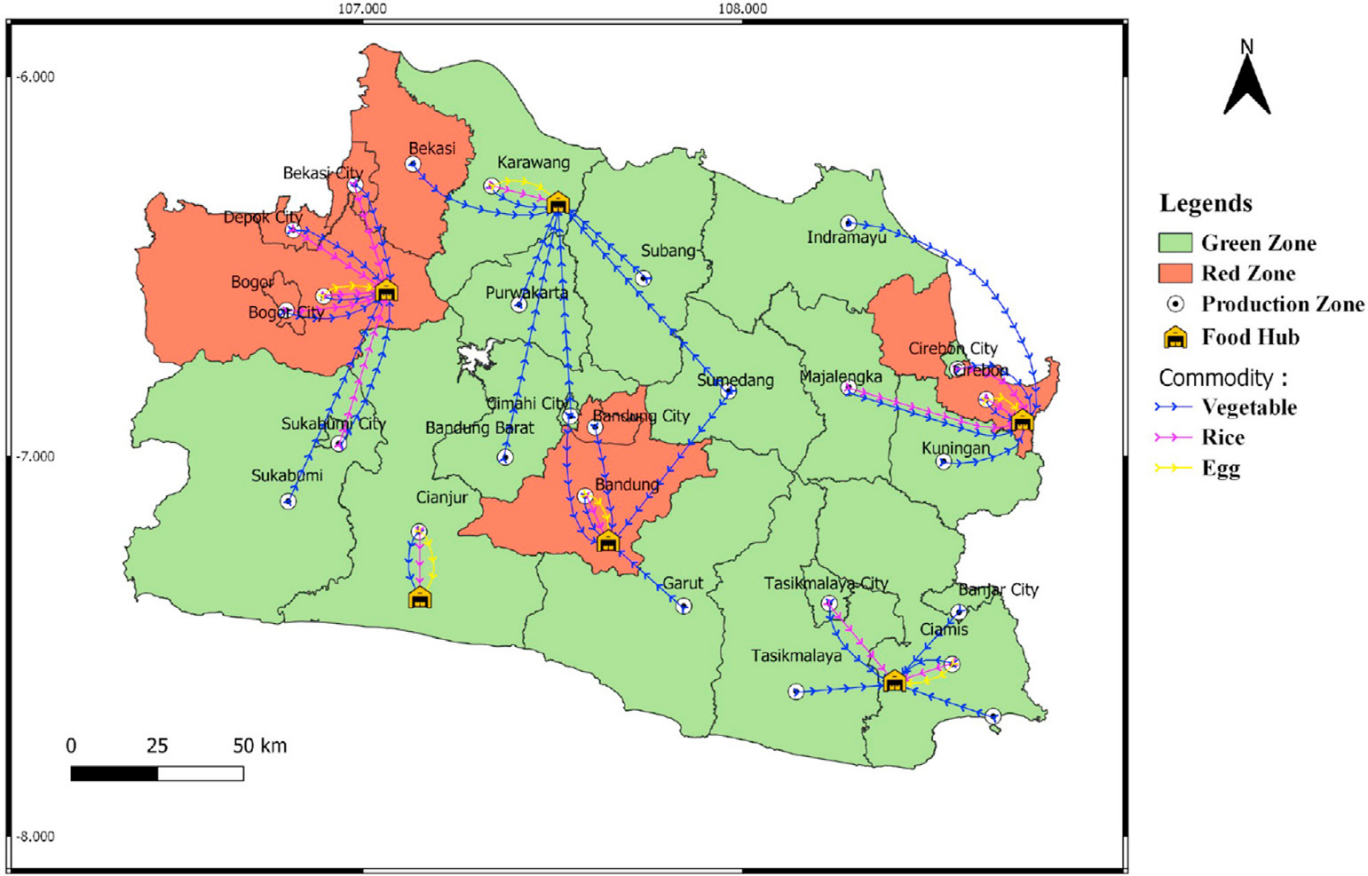
Food supply network from production zone to RFH for case 1 in Scenario 3.

Each production area can supply crops to the nearest RFH with due consideration to hygiene protocols as a preventive measure for COVID-19 transmission. The food supply network with a system based on normal conditions ensures the availability of food for the communities, as presented in Figure 9. This condition can have implications for social and economic factors of the communities in urban and rural areas.

**Figure 10:**
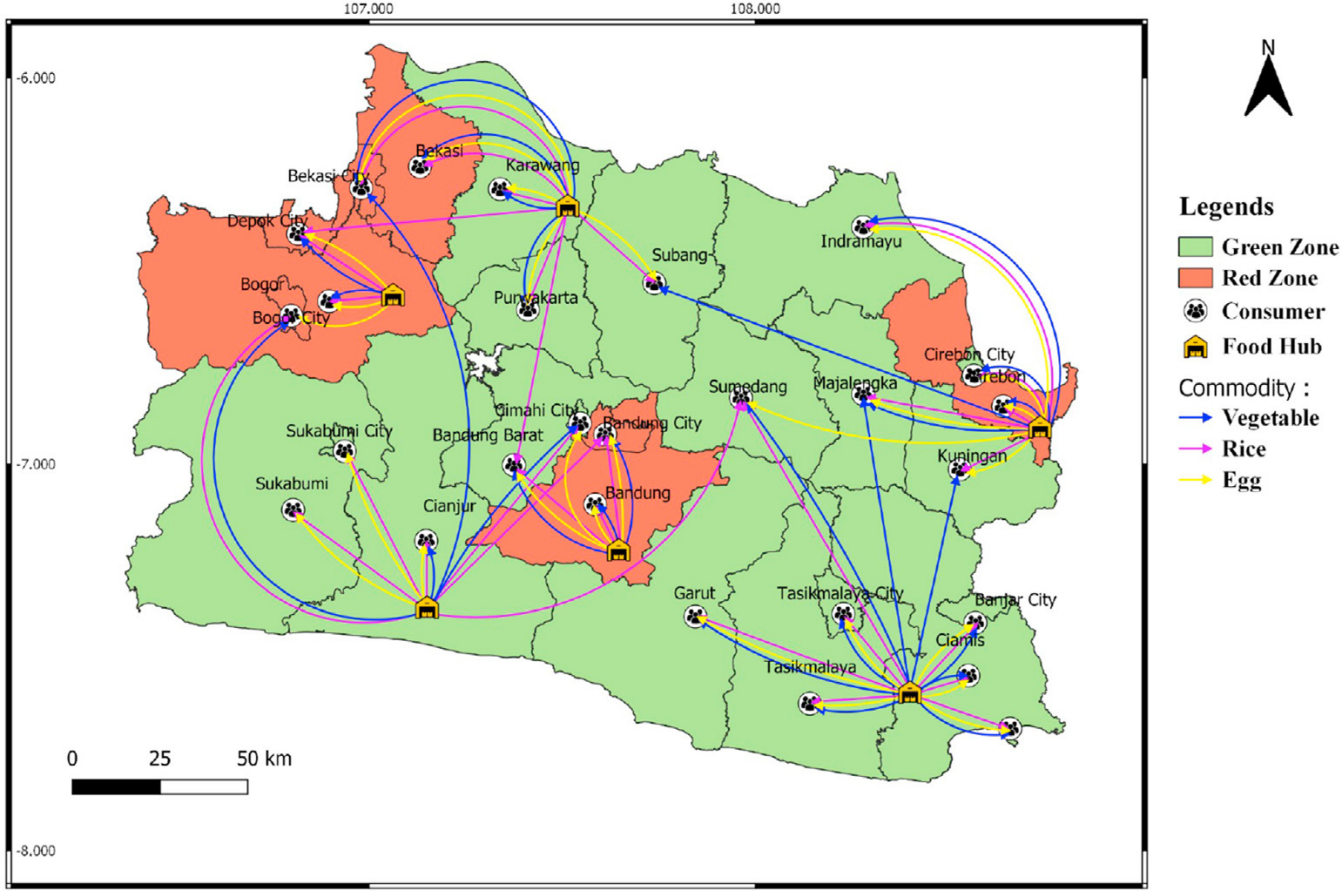
Food supply network from RFH to consumer for case 1 in scenario 3.

In distributing food from RFHs to consumers, RFH services cover all areas within a certain distance, as presented in Figure 10. Food distribution from RFHs is done through traditional markets, modern markets, cafés, restaurants, catering services, hospitals, hotels, and online market places. The distribution system guarantees the availability and traceability of food, making it easy to control food security in each consumer area.

### 4.5 Discussion of optimal scenarios

As a result of the COVID-19 pandemic, the issue of the food system has emerged. The food system includes all aspects of providing food. The implementation of the large-scale social distancing impacted the uncertainty of food demand and supply. Therefore, it is essential to develop a food system to tackle the uncertainty of food supply. In this paper, a model was developed that focuses on the RFH location and network. It determined the improvement of food security for society by considering aspects of sustainability, food safety, and effectiveness of the food supply network. A set of rules were applied to each situation to arrive at the best scenario for food supply during the pandemic. The three scenarios are compared and summarized in Table 19.

**Table 19:** Comparison of the three scenarios.

Scenarios	Total Logistics Cost (Rp/kg)		Fulfillment Ratio (%fulfilled)						Total RFH	
			Rice		Chicken Egg		Vegetable		Built	
	Case 1	Case 2	Case 1	Case 2	Case 1	Case 2	Case 1	Case 2	Case 1	Case 2
Scenario 1	5,211.97	5,212.89	82.33%	90.75%	100%	100%	71.88%	77.74%	11	17
Scenario 2	5,211.35	5,214.36	100%	100%	100%	100%	92.29%	77.74%	11	16
Scenario 3	5,208.38	5,208.38	100%	100%	100%	100%	92.29%	92.29%	6	6

Scenario 1 implements large-scale social distancing so that areas designated as red zones cannot deliver or receive food supplies from other regions and vice versa. The limitation in this scenario aims to minimize the spread of the disease. This scenario becomes a problem if the red zone only falls in urban areas that do not have sufficient capacity for food production. The number of RFHs in scenario 1 is higher than in both scenarios 2 and 3. The results for this scenario will also worsen when the number of areas classified as red zones increases, as each location will need to build RFHs and fulfill food needs independently. A change in the number of red zones also affects the total cost of RFH development. The application of large-scale social distancing on the food supply network inhibits economic circulation and social activities. This condition is not in line with the principle of food security and sustainability (Sharpe et al., 2020).

Scenario 2 applies partial social distancing; other zones support the food needs of the red zone and are predicted to decrease hunger. Food distribution activities from the other areas are also carried out by implementing food and health safety protocols recommended by the government as a way to minimize the spread of the disease. Food security should consider the spreading of the virus between producers, retailers, and customers and avoid massive food insecurity.

Scenario 2 aims at keeping the food supply network functioning to be able fulfill food needs. However, this scenario will only work well if all red zones are in urban areas. The situation would be different if the red zone extends into the production center, causing a shortage of food supply in several areas. It is feared that this situation will worsen the community's physical and mental health (Highlander and Singley, 2020). Moreover, the costs required for RFH development are not much different from scenario 1.

In scenario 3, food delivery is carried out under the new era in the food supply chain, which involves the implementation of health and food safety protocols. Each production area can supply the crops to the nearest RFH, and RFHs can serve the entire area based on their range and capacity. The vegetable needs can be fulfilled by substituting other vegetable commodities not included in this model's calculation. RFH development in scenario 3 is not affected by the increase in the number of red zones and location changes. Therefore, the costs required for RFH development is lower than in scenarios 1 and 2. The RFH could be the optimal form of food supply network as it can ensure the supply of food for the entire region, and also has implications for reducing carbon emissions (Galanakis, 2020; Hobbs, 2020; Sharpe et al., 2020).

The determination of the scenario takes into account aspects of the food system, sustainability, and health regulations (Hobbs, 2020; Sharpe et al., 2020; Djalante et al., 2020; Brand et al., 2019; Mittal et al., 2018; Brinkley, 2018; Ge et al., 2018; Levkoe et al., 2018; Dasaklis et al., 2012). The food system should be developed with consideration of food security and food safety (Galanakis, 2020; Rizou et al., 2020). In scenario 3, RFH is designed to benefit all levels of society from the availability of sufficient food. Upstream actors can minimize overproduction to increase production efficiency and value addition (Kharisma and Perdana, 2019; Barba et al., 2015). Downstream actors are becoming interested in local foods, including perceptions about sustainability, hygiene, and health benefits (Hobbs, 2020; Galanakis, 2020; Rizou et al., 2020).

Food security during the pandemic must be ensured. Maintaining hygiene during production and logistics, as well as the hygiene of food and workers are important considerations while ensuring food security to the whole community during a pandemic (WHO, 2020; Rizou et al., 2020; Galanakis, 2018). Therefore, RFHs could be used as a resilient food network to ensure the availability and safety of food (Béné, 2020; Galanakis, 2020; Rizou et al., 2020).

From the perspective of sustainability, food delivery can reduce carbon emissions from efficient use of vehicle fuel, decreased food loss during distribution and post-harvest, and reduction of food waste during the selling process (Hakovirta and Denuwara, 2020; Nagarajan et al., 2019; Galanakis et al., 2018). RFHs are designed based on the locality of agricultural products and aim to improve the social and economic condition of communities affected by the pandemic (Nicola et al., 2020; Phillipson et al., 2020). Therefore, the development of RFHs needs to be strengthened by the development of efficient and interconnected food chain networks (Galanakis, 2020; Handayati et al., 2015).

To reduce the spread of COVID-19 and support physical distancing, digital food services could be adopted to optimize the RFH services. Such services will have a longer-term effect on the food supply chain, including the increase of e-commerce and customer preference on the local food supply network (Hobbs, 2020; Keesara et al., 2020). Digital food services could also reduce food loss and waste, make a traceability system possible, fulfill the nutrition requirements of the customer, as well as provide a fast response to imminent economic crises in the era of the COVID-19 pandemic (Sarfarazi et al., 2020). Functional local food can fortify health and improve the consumer's immunity system (Galanakis, 2020).

The conception of the model also considers the policies that apply both in West Java Province and the central government (Manganelli et al., 2020). An alternative model of RFH development in West Java Province can be a reference for the government to build a food supply network that emphasizes aspects of food safety and health based on the specific conditions in West Java Province.

## 5 Conclusion and future research

The best scenario is scenario 3, as it illustrates ideal conditions during typical situations. This scenario is chosen because none of the RFHs have territorial boundaries (red and green zones) during the procurement. The food supply network's delivery should conform to health and safety protocols to minimize the spread of disease. The number of RFH that should build is fixed, that is, six RFHs, and so the investment cost of RFH development is lower than in scenarios 1 and 2. The RFHs and food supply network are in a strategic location, food is supplied from the nearest RFH region, and RFH serves the closest coverage area. This scenario has an impact on the efficiency of distance and the use of diesel fuel, which leads to an increase in farmers' income and customers' access to local food.

A possible topic for future research is minimizing the spread of disease by developing digital services. Digital food services could be an alternative technology to decrease human interaction, reduce the time spent in product purchase, decrease carbon emissions, reduce food loss and waste, and maximize warehouse capacity. Some food processing technologies to process food waste from the supply chain could also be considered. Digitalization is useful in obtaining bioactive ingredients to strengthen the consumer's immune system and minimize food waste for a more sustainable food supply chain. The model scenario presented here could be used as a reference for the Provincial Government to build RFH during the COVID-19 pandemic and as a recommended strategy to develop food security in the new era.

## Declarations

### Author contribution statement

T. Perdana: Conceived and designed the experiments; performed the experiments; analyzed and interpreted the data; wrote the paper D. Chaerani and A. L. H. Achmad: Performed the experiments; analyzed and interpreted the data; contributed reagent, materials, analysis tools or data; wrote the paper F. R. Hermiatin: Performed the experiments; analyzed and interpreted the data; and wrote the paper.

### Funding statement

This research was funded by Indonesian The Ministry of Research and Technology/National Research and Innovation Agency (1827/UN6.3.1/LT/2020).

### Competing interest statement

The authors declare no conflict of interest.

### Additional information

No additional information is available for this paper.

## Footnotes

1 Red zones are the worst-affected areas during the pandemic and with many more casualties than orange, yellow, blue, and green zones (Shaker et al., 2020).

## References

[bib1] Ballantyne-Brodie E., Telalbasic I. (2017). Designing local food systems in everyday life through service design strategies. Des. J..

[bib2] Barba F.J., Galanakis C.M., Esteve M.J., Frigola A., Vorobiev E. (2015). Potential use of pulsed electric technologies and ultrasounds to improve the recovery of high-added value compounds from blackberries. J. Food Eng..

[bib3] Barham J., Tropp D., Enterline K., Farbman J., Fisk J., Kiraly S. (2012). Regional Food Hub Resource Guide.

[bib4] Ben-Tal A., El Ghaoui L., Nemirovski A. (2009).

[bib5] Ben-Tal A., Nemirovski A. (2002). Robust optimization–methodology and applications. Math. Program..

[bib6] Béné C. (2020). Resilience of Local Food Systems and Links to Food Security–A Review of Some Important Concepts in the Context of Covid-19 and Other Shocks.

[bib7] Berno T. (2017). Social enterprise, sustainability and community in post-earthquake Christchurch. Journal of Enterprising Communities: People and Places in the Global Economy.

[bib8] Black C., Moon G., Baird J. (2015). Dietary inequalities: what is the evidence for the effect of the neighborhood food environment?. Health Place.

[bib9] Blay-Palmer A., Landman K., Knezevic I., Hayhurst R. (2013). Constructing Resilient, Transformative Communities through Sustainable "food Hubs".

[bib10] Brand D., Nicholson H., Allen N. (2019). The Role of Placemaking as a Tool for Resilience: Case Studies from Post-Earthquake Christchurch, New Zealand.

[bib11] Brinkley C. (2018). The small world of the alternative food network. Sustainability.

[bib12] Chen Y.-h., Chen M.-x., Mishra A.K. (2020). Subsidies under uncertainty: modeling of input and output-oriented policies. Econ. Modell..

[bib13] Cleveland D.A., Müller N.M., Tranovich A.C., Mazaroli D.N., Hinson K. (2014). Local food hubs for alternative food systems: a case study from Santa Barbara county, California. J. Rural Stud..

[bib14] Dasaklis T.K., Pappis C.P., Rachaniotis N.P. (2012). Epidemics control and logistics operations: a review. Int. J. Prod. Econ..

[bib15] Deng Q., Zinoviadou K.G., Galanakis C.M., Orlien V., Grimi N., Vorobiev E., Lebovka N., Barba F.J. (2015). The effects of conventional and non-conventional processing on glucosinolates and its derived forms, isothiocyanates: extraction, degradation, and applications. Food Eng. Rev..

[bib16] Devereux S., Béné C., Hoddinott J. (2020). Conceptualizing Covid-19's Impacts on Household Food Security.

[bib17] Djalante R., Lassa J., Setiamarga D., Mahfud C., Sudjatma A., Indrawan M., Haryanto B., Sinapoy M.S., Rafliana I., Djalante S. (2020). Review and analysis of current responses to covid-19 in Indonesia: period of January to march 2020. Progr. Disaster Sci..

[bib18] Etemadnia H., Goetz S.J., Canning P., Tavallali M.S. (2015). Optimal wholesale facilities location within the fruit and vegetables supply chain with bimodal transportation options: an lp-mip heuristic approach. Eur. J. Oper. Res..

[bib19] Farmer J.R., Betz M.E. (2016). Rebuilding local foods in Appalachia: variables affecting distribution methods of West Virginia farms. J. Rural Stud..

[bib20] Fischer M., Pirog R., Hamm M.W. (2015). Food hubs: definitions, expectations, and realities. J. Hunger Environ. Nutr..

[bib21] Galanakis C.M. (2012). Recovery of high added-value components from food wastes: conventional, emerging technologies and commercialized applications. Trends Food Sci. Technol..

[bib22] Galanakis C.M. (2013). Emerging technologies for the production of nutraceuticals from agricultural by-products: a viewpoint of opportunities and challenges. Food Bioprod. Process..

[bib23] Galanakis C.M. (2015). Separation of functional macromolecules and micromolecules: from ultrafiltration to the border of nanofiltration. Trends Food Sci. Technol..

[bib24] Galanakis C.M. (2018). Phenols recovered from olive mill wastewater as additives in meat products. Trends Food Sci. Technol..

[bib25] Galanakis C.M. (2020). The food systems in the era of the coronavirus (covid-19) pandemic crisis. Foods.

[bib26] Galanakis C.M., Tsatalas P., Galanakis I.M. (2018). Implementation of phenols recovered from olive mill wastewater as uv booster in cosmetics. Ind. Crop. Prod..

[bib27] Ge H., Goetz S., Canning P., Perez A. (2018). Optimal locations of fresh produce aggregation facilities in the United States with scale economies. Int. J. Prod. Econ..

[bib28] Gorissen B.L., Yanıkoğlu İ., den Hertog D. (2015). A practical guide to robust optimization. Omega.

[bib29] Gray R.S. (2020). Agriculture, transportation, and the covid-19 crisis. Canadian J. Agricult. Econom./Revue Canadienne d‘agroeconomie.

[bib30] Gregorioa G.B., Ancog R.C. (2020). Assessing the impact of the covid-19 pandemic on agricultural production in Southeast Asia: toward transformative change in agricultural food systems. Asian J. Agricult. Dev..

[bib31] Hakovirta M., Denuwara N. (2020). How Covid-19 Redefines the Concept of Sustainability.

[bib32] Handayati Y., Simatupang T.M., Perdana T. (2015). Value co-creation in agri-chains network: an agent-based simulation. Proc. Manufact..

[bib33] Hiassat A., Diabat A., Rahwan I. (2017). A genetic algorithm approach for location-inventory-routing problem with perishable products. J. Manuf. Syst..

[bib34] Highlander H., Singley A. (2020). Covid-19: a mathematical model for the effect of social distancing on the spread of covid-19. Lett. Biomath..

[bib35] Hilmers A., Hilmers D.C., Dave J. (2012). Neighborhood disparities in access to healthy foods and their effects on environmental justice. Am. J. Publ. Health.

[bib36] Hobbs J.E. (2020). Food supply chains during the covid-19 pandemic. Canadian J. Agricult. Econom./Revue Canadienne d‘agroeconomie.

[bib37] Johnson R., Aussenberg R.A., Cowan T. (2012). The Role of Local Food Systems in US Farm Policy.

[bib38] Keesara S., Jonas A., Schulman K. (2020). Covid-19 and health care’s digital revolution. N. Engl. J. Med..

[bib39] Kharisma A., Perdana T. (2019). Linear programming model for vegetable crop rotation planning: a case study. Int. J. Agric. Resour. Govern. Ecol..

[bib40] Kotani H., Yokomatsu M., Ito H. (2020). Potential of a shopping street to serve as a food distribution center and an evacuation shelter during disasters: case study of Kobe, Japan. Int. J. Disaster Risk Reduct..

[bib42] Le Velly R., Dufeu I. (2016). Alternative food networks as "market agencementse": exploring their multiple hybridities. J. Rural Stud..

[bib43] Levkoe C.Z., Hammelman C., Craven L., Dandy G., Farbman J., Harrison J., Mount P. (2018). Building sustainable communities through food hubs. J. Agricult. Food Syst. Commun. Develop..

[bib44] Liu M., Cao J., Liang J., Chen M. (2020). Epidemic-logistics Modeling: A New Perspective on Operations Research.

[bib45] Manganelli A., Van den Broeck P., Moulaert F. (2020). Socio-political dynamics of alternative food networks: a hybrid governance approach. Territory Pol. Govern..

[bib46] Mitchell R., Maull R., Pearson S., Brewer S., Collison M. (2020). The Impact of Covid-19 on the UK Fresh Food Supply Chain.

[bib47] Mittal A., Krejci C.C. (2019). A hybrid simulation modeling framework for regional food hubs. J. Simulat..

[bib48] Mittal A., Krejci C.C., Craven T.J. (2018). Logistics best practices for regional food systems: a review. Sustainability.

[bib49] Mukhamedjanova K. (2020). The impact of the covid-19 pandemic on the supply chain of agricultural products. Asian J. Technol. Manag. Res..

[bib50] Nagarajan J., Krishnamurthy N.P., Ramanan R.N., Raghunandan M.E., Galanakis C.M., Ooi C.W. (2019). A facile water-induced complexation of lycopene and pectin from pink guava byproduct: extraction, characterization and kinetic studies. Food Chem..

[bib51] Nicola M., Alsafi Z., Sohrabi C., Kerwan A., Al-Jabir A., Iosifidis C., Agha M., Agha R. (2020). The socio-economic implications of the coronavirus and covid-19 pandemic: a review. Int. J. Surg..

[bib52] O‘Hara S. (2015). Food security: the urban food hub solution. Solutions.

[bib53] Pertamina (2018). Daftar Harga Bbk Tmt 10 Oktober 2018 - Pt Pertamina Persero. https://www.pertamina.com/id/news-room/announcement/daftar-harga-bbk-tmt-10-oktober-201.

[bib54] Pertamina (2018). Daftar Harga Bbk Tmt 24 Februari 2018 - Pt Pertamina Persero. https://www.pertamina.com/id/news-room/announcement/daftar-harga-bbk-tmt-24-februari-2018.

[bib55] Pertamina (2018). Daftar Harga Bbk Tmt 24 Maret 2018 - Pt Pertamina Persero. https://www.pertamina.com/id/news-room/announcement/daftar-harga-bbk-tmt-24-maret-2018.

[bib56] Phillipson J., Gorton M., Turner R., Shucksmith M., Aitken-McDermott K., Areal F., Cowie P., Hubbard C., Maioli S., McAreavey R. (2020). The covid-19 pandemic and its implications for rural economies. Sustainability.

[bib57] Prost S., Crivellaro C., Haddon A., Comber R. (2018). Food democracy in the making: designing with local food networks. Proceedings of the 2018 CHI Conference on Human Factors in Computing Systems.

[bib58] Pulighe G., Lupia F. (2020). Food first: covid-19 outbreak and cities lockdown a booster for a wider vision on urban agriculture. Sustainability.

[bib59] Rao S.S. (2019). Engineering Optimization: Theory and Practice.

[bib60] Rizou M., Galanakis I.M., Aldawoud T.M., Galanakis C.M. (2020). Safety of foods, food supply chain and environment within the covid-19 pandemic. Trends Food Sci. Technol..

[bib61] Robinson C., Shirazi A., Liu M., Dilkina B. (2016). Network optimization of food flows in the us. 2016 IEEE International Conference on Big Data (Big Data).

[bib62] Rose N. (2017). Community food hubs: an economic and social justice model for regional Australia?. Rural Soc..

[bib63] Sarfarazi M., Jafari S.M., Rajabzadeh G., Galanakis C.M. (2020). Evaluation of microwave-assisted extraction technology for separation of bioactive components of saffron (crocus sativus l.). Ind. Crop. Prod..

[bib64] Shaker M.S., Oppenheimer J., Grayson M., Stukus D., Hartog N., Hsieh E.W., Rider N., Dutmer C.M., Vander Leek T.K., Kim H. (2020). Covid-19: pandemic contingency planning for the allergy and immunology clinic. J. Allergy Clin. Immunol.: In Pract..

[bib65] Sharpe P.A., Bell B.A., Liese A.D., Wilcox S., Stucker J., Hutto B.E. (2020). Effects of a food hub initiative in a disadvantaged community: a quasi-experimental evaluation. Health Place.

[bib66] Silver M., Bediako A., Capers T., Kirac A., Freudenberg N. (2017). Creating integrated strategies for increasing access to healthy affordable food in urban communities: a case study of intersecting food initiatives. J. Urban Health.

[bib67] Sonnino R., Torres C.L., Schneider S. (2014). Reflexive governance for food security: the example of school feeding in Brazil. J. Rural Stud..

[bib68] Stanimirovic I.P. (2012). Compendious lexicographic method for multi-objective optimization. Facta Univ. – Ser. Math. Inf..

[bib69] Statistics Indonesia (2020). Indonesia Population Projection 2010-2035.

[bib70] Statistics of Jawa Barat (2009). Jawa Barat Province in Figures 2009.

[bib71] Statistics of Jawa Barat (2010). Jawa Barat Province in Figures 2010.

[bib72] Statistics of Jawa Barat (2011). Jawa Barat Province in Figures 2011.

[bib73] Statistics of Jawa Barat (2012). Jawa Barat Province in Figures 2012.

[bib74] Statistics of Jawa Barat (2013). Jawa Barat Province in Figures 2013.

[bib75] Statistics of Jawa Barat (2014). Jawa Barat Province in Figures 2014.

[bib76] Statistics of Jawa Barat (2015). Jawa Barat Province in Figures 2015.

[bib77] Statistics of Jawa Barat (2016). Jawa Barat Province in Figures 2016.

[bib78] Statistics of Jawa Barat (2017). Jawa Barat Province in Figures 2017.

[bib79] Statistics of Jawa Barat (2018). Jawa Barat Province in Figures 2018.

[bib80] Tang O., Lau Y.Y. (2013). Logistics aspects of avian influenza pandemic in Hong Kong. Int. J. Logist. Syst. Manag..

[bib81] Walker R.E., Keane C.R., Burke J.G. (2010). Disparities and access to healthy food in the United States: a review of food deserts literature. Health Place.

[bib82] Wang S., Tao F., Shi Y. (2018). Optimization of location–routing problem for cold chain logistics considering carbon footprint. Int. J. Environ. Res. Publ. Health.

[bib83] WHO (2020). Covid-19 and Food Safety: Guidance for Food Businesses: Interim Guidance, 07 April 2020.

[bib84] Yanıkoğlu İ., Gorissen B.L., den Hertog D. (2019). A survey of adjustable robust optimization. Eur. J. Oper. Res..

[bib85] Zinoviadou K.G., Galanakis C.M., Brnčić M., Grimi N., Boussetta N., Mota M.J., Saraiva J.A., Patras A., Tiwari B., Barba F.J. (2015). Fruit juice sonication: implications on food safety and physicochemical and nutritional properties. Food Res. Int..

